# Generation of Novel Monoclonal Antibodies Recognizing Rabbit CD34 Antigen

**DOI:** 10.3390/biom15071021

**Published:** 2025-07-15

**Authors:** Jaromír Vašíček, Miroslav Bauer, Eva Kontseková, Andrej Baláži, Andrea Svoradová, Linda Dujíčková, Eva Tvrdá, Jakub Vozaf, Peter Supuka, Peter Chrenek

**Affiliations:** 1 NPPC—Research Institute for Animal Production Nitra, Hlohovecká 2, 951 41 Lužianky, Slovakia; miroslav.bauer@nppc.sk (M.B.); andrej.balazi@nppc.sk (A.B.); andrea.svoradova@nppc.sk (A.S.); linda.dujickova@nppc.sk (L.D.); jakub.vozaf@nppc.sk (J.V.); peter.chrenek@uniag.sk (P.C.); 2 Institute of Biotechnology, Faculty of Biotechnology and Food Science, Slovak University of Agriculture in Nitra, Tr. A. Hlinku 2, 949 76 Nitra, Slovakia; eva.tvrda@uniag.sk; 3 Department of Botany and Genetics, Faculty of Natural Sciences, Constantine the Philosopher University in Nitra, Nábrežie Mládeže 91, 949 74 Nitra, Slovakia; 4 Institute of Neuroimmunology, Slovak Academy of Sciences, Dúbravská cesta 9, 845 10 Bratislava, Slovakia; eva.kontsekova@savba.sk; 5 VETSERVIS Ltd., Kalvária 3, 949 01 Nitra, Slovakia; supuka.peter@gmail.com

**Keywords:** rabbit CD34, hematopoietic stem/progenitor cells, hybridoma technology, monoclonal antibodies, flow cytometry

## Abstract

The rabbit is a widely used experimental model for human translational research and stem cell therapy. Many studies have focused on rabbit mesenchymal stem cells from different biological sources for their possible application in regenerative medicine. However, a minimal number of studies have been published aimed at rabbit hematopoietic stem/progenitor cells, mainly due to the lack of specific anti-rabbit CD34 antibodies. In general, CD34 antigen is commonly used to identify and isolate hematopoietic stem/progenitor cells in humans and other animal species. The aim of this study was to develop novel monoclonal antibodies highly specific to rabbit CD34 antigen. We used hybridoma technology, two synthetic peptides derived from predicted rabbit CD34 protein, and a recombinant rabbit CD34 protein as immunogens to produce monoclonal antibodies (mAbs) specific to rabbit CD34. The produced antibodies were screened for their binding activity and specificity using ELISA, flow cytometry, and Western blot analysis. Finally, four mAbs (58/47/26, 58/47/34, 182/7/80, and 575/36/8) were selected for the final purification process. The purified mAbs recognized up to 2–3% of total rabbit bone marrow cells, while about 2% of those cells exhibited CD45 expression, which are likely rabbit primitive hematopoietic stem cells and their hematopoietic progenitors, respectively. The newly generated and purified mAbs specifically recognize CD34 antigen in rabbit bone marrow or peripheral blood and can be therefore used for further immunological applications, to study rabbit hematopoiesis or to establish a new animal model for hematopoietic stem cell transplantation studies.

## 1. Introduction

In general, if a foreign molecule enters the organism’s body, the immune system immediately responds by producing antibodies. Such a foreign molecule could be an enzyme, hormone, various proteins, any bacterial structure, a virus, or even a eukaryotic cell, and all are called antigens. The immune system recognizes the foreign molecular structure and selectively produce antibodies, which specifically bind only to certain antigens [1,2]. Antibodies are glycoproteins, also known as immunoglobulins, produced by B-cells. Antibodies are composed of two structural units, a heavy and a light chain. In addition, each heavy chain consists of one variable and three constant regions. On the other hand, one variable and one constant region are present in each light chain. In fact, the variable regions are crucial for the recognition mechanism of antigens. The antigen-binding fragments are attached to the crystallizable fragments (Fc), also called Fc regions, of the antibody heavy chains. According to the structure of the Fc regions, five different antibody isotypes are recognized, i.e., IgM, IgG, IgA, IgD, and IgE. The IgG isotypes are the most common and smallest antibody isotypes and thus offer the greatest therapeutical use [1,3].

Generally, two types of antibodies can be distinguished, i.e., monoclonal and polyclonal antibodies, depending on their specificity. Monoclonal antibodies (mAbs) are monospecific antibodies produced by a single B-cell clone, with high specificity and affinity to one antigen epitope [4]. The specific mAb can be produced by hybridoma technology, which was originally established by Kohler and Milstein in 1975 [5]. However, this technology remains currently important and is commonly used to generate mAbs specific to any epitope presented on an antigen or any other immunogen. The produced mAbs have become powerful and highly specific molecular recognition tools in various research fields such as biochemistry, molecular biology, medicine, etc. [3].

The hybridoma technology using mice to produce monoclonal antibodies comprises a complex process consisting of several steps. It is generally based on the natural ability of any animal to generate fully functional mAbs with high specificity and affinity. The first step is to develop and optimize a specific antigen with sufficient immunogenicity. Afterwards, the optimized antigen is used for the immunization of the host animal, in combination with a suitable adjuvant, which can last for several weeks. Blood samples of the immunized animals are then tested for the presence of antibodies in their sera to determine the antibody concentration, specificity, and reactivity to the immunizing antigen throughout the immunization procedure. Only mice showing high titers of specific antibodies were selected for the subsequent isolation of B-cells from their spleens [1,6,7]. Hybridomas are usually produced by the fusion of mice B-cells derived from animals immunized with an antigen and immortal cancer cells, e.g., myeloma cells, which lack hypoxanthine guanine phosphoribosyltransferase (HGPRT). This fusion resulted in hybrid cell line with immortal properties and limitless antibody production [1]. The process of hybridoma generation requires culturing in specific medium containing xanthine-aminopterin-thymidine (HAT), which causes the death of unfused myeloma cells. Moreover, the unfused B-cells also die, as they cannot proliferate in vitro. At last, only the hybridoma cell lines remain viable and successfully proliferate in HAT medium. Supernatants from growing hybridomas are subsequently screened for the produced mAbs using the ELISA method. Only positive hybridomas are further sub-cloned for stable antibody production [8]. Additionally, the created hybridoma cell lines can be cryopreserved for later use.

Many useful mouse monoclonal antibodies have been successfully produced by hybridoma technology over the years [8]. They can be used for the detection, purification, or characterization of any target cell, tissue, or substance by different applications, e.g., flow cytometry, magnetic-activated cell sorting, immunological arrays, therapeutic applications, etc. [3,9]. Such very useful mAbs have been generated against human CD34 antigen, which is commonly used to identify and isolate human hematopoietic stem and progenitor cells for further therapeutical procedures. CD34 itself is a cell surface protein expressed mainly on stem and progenitor cells and mature vascular endothelial tissue. However, as the hematopoietic stem cells and progenitors differentiate into mature cells, they basically cease CD34 expression. For that reason, cells expressing CD34 represent a very rare cell population within the adult organism [10]. Different animal models have been used for the study of hematopoietic stem cells and their transplantation [11,12,13,14]. Some of these studies used cross-reactive antibodies, if an animal-specific antibody was not available. On the other hand, specific mAbs have been already developed for some animal species using hybridoma technology to detect CD34 antigen [15,16,17].

Rabbit is also a common animal model for the study of human diseases, not only due to the fact that rabbits suffer from the same hereditary diseases as humans [18], but they are even genetically and physiologically closer to primates than are rodents [19,20,21]. Rabbits have previously been used for orthopedic and cardiovascular surgery studies, as well as for the study of neoplastic diseases and other conditions [22]. In addition, rabbit stem cells are widely used in regenerative medicine and tissue engineering, such as for bone, cartilage, and nerve regeneration; to model lung, cardiovascular, and metabolic human diseases; to test new potential therapies; to create functional organs or tissues for further transplantation; to study how to deliver stem cells for therapeutic purposes; or for the study of cell differentiation mechanisms and factors [23,24]. In general, rabbit stem cells can help accelerate the development of stem cell therapies for the treatment of serious human diseases via translational research [25]. However, according to our previous study, commercially available CD34 antibodies exhibit low cross-reactivity with rabbit cells [26], which makes it impossible to use rabbit hematopoietic CD34-positive cells for further translational research. In addition, as the pure line of rabbit hematopoietic stem/progenitor cells has never been isolated, it was impossible to use a native cell line as a potential antigen for mice immunization to obtain rabbit-specific CD34 antibodies. On the other hand, synthetic peptides or recombinant proteins are commonly used as the immunogen to produce antigen-specific antibodies, such as in the case of mAbs generated against canine, porcine, or ovine CD34 [15,16,17]. Therefore, the aim of the presented study was to develop novel monoclonal antibodies highly specific to rabbit CD34 antigen via the immunization of mice with two synthetic peptides and/or a recombinant protein designed from the extracellular domain of the predicted rabbit CD34 protein and subsequent hybridoma technology.

## 2. Materials and Methods

### 2.1. Animals and Cell Isolation

Young (3–8 months old) and healthy rabbits of the New Zealand White (NZW) line (*n* = 12) and Holic blue (HB) line (*n* = 3), reared at the NPPC—Research Institute for Animal Production Nitra (NPPC-RIAP Nitra), as described previously [26], were used for the flow cytometric analyses and Western blot experiments. Six-week-old female (*n* = 9) BALB/c mice (VELAZ s.r.o., Prague, Czech Republic) were used for immunization and the development of mouse mAbs. The immunization of mice was carried out according to institutional animal care guidelines conforming to international standards and was approved by the State Veterinary and Food Committee of the Slovak Republic (Ro-1-3/2020-220) and by the Ethics Committee of the Institute of Neuroimmunology, Slovak Academy of Science, Bratislava. All additional work was performed with the approval of the Ministry of Agriculture and Rural Development of the Slovak Republic, no. SK U 18021, in accordance with the ethical guidelines presented in the Slovak Animal Protection Regulations (RD 377/12), which conforms to the Code of Ethics of the EU Directive 2010/63/EU for animal experiments.

Peripheral blood and bone marrow mononuclear cells (PBMCs and BMMCs, respectively) were collected from humanely sacrificed rabbits using Biocoll solution (Biochrom, Berlin, Germany) and density-gradient centrifugation, as described previously [26,27]. Freshly isolated PBMCs and BMMCs were immediately applied for flow-cytometry to analyze the specific binding of newly produced mAbs. Simultaneously, aliquots of fresh cell samples were lysed in SDS sample buffer and frozen at −80 °C for further Western blot analysis.

### 2.2. Analysis of Amino Acid Sequence of Rabbit CD34 Protein and Peptide Synthesis

**Figure 1:**
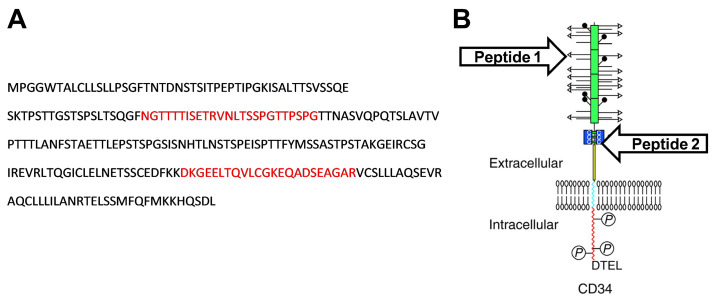
Peptide sequences and their estimated localization. Predicted immunogenic oligopeptides in red (**A**) and rough estimation of their localization in the CD34 protein structure (**B**). CD34 protein structure was adopted and modified from Nielsen & McNagny [29].

According to the UniProt database, the predicted sequence of rabbit CD34 protein (G1SJT2) consist of 412 amino acids [28], which is identical to amino acid sequences derived from rabbit CD34 mRNA transcript variants X1 and X2 (NCBI Reference Sequence: XM_008268471.3 and XM_008268472.3, respectively). The extracellular domain of rabbit CD34 protein (256 amino acids: MPGGWTALCLLSLLPSGFTNTDNSTSITPEPTIPGKISALTTSVSSQESKTPSTTGSTSPSLTSQGFNGTTTTISETRVNLTSSPGTTPSPGTTNASVQPQTSLAVTVPTTTLANFSTAETTLEPSTSPGSISNHTLNSTSPEISPTTFYMSSASTPSTAKGEIRCSGIREVRLTQGICLELNETSSCEDFKKDKGEELTQVLCGKEQADSEAGARVCSLLLAQSEVRAQCLLLILANRTELSSMFQFMKKHQSDL), predicted according to the protein structure of the CD34 family [29], was used to design immunogenic oligopeptides using B-cell epitope probability prediction software (http://tools.iedb.org/bcell/ (accessed on 13 February 2019); [30]). The designed peptides, peptide 1 (NGTTTTISETRVNLTSSPGTTPSPG) and peptide 2 (DKGEELTQVLCGKEQADSEAGAR), were commercially synthesized (CASLO ApS, Kongens Lyngby, Denmark) and used for monoclonal antibody generation (Figure 1).

### 2.3. Expression and Purification of Recombinant Rabbit CD34 Protein

**Figure 2:**
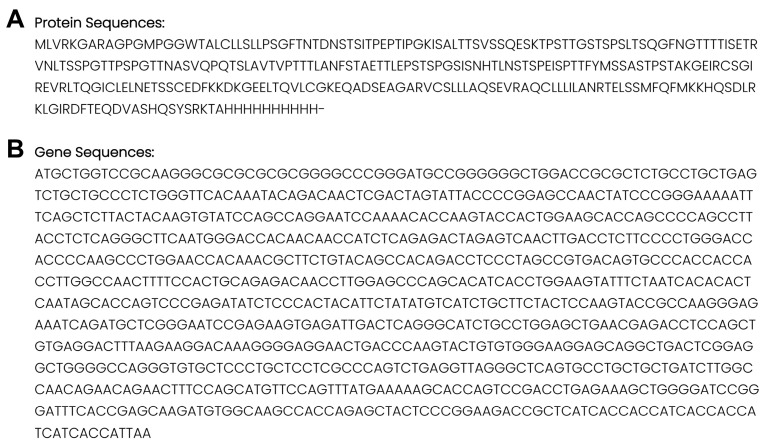
Protein and gene sequence of recombinant rabbit CD34. Amino acid sequence (**A**) of designed recombinant rabbit CD34 protein with His-tag and nucleotide sequence (**B**) of rabbit CD34 gene used for expression of recombinant rabbit CD34 protein representing the extracellular domain of predicted rabbit CD34.

The recombinant rabbit CD34 protein (recCD34) was commercially produced by Protein Expression & Purification Service of Sino Biological Inc. (Beijing, China) using a proprietary procedure of HEK293 transient expression (Figure 2). Briefly, the target gene was amplified by PCR and inserted into the corresponding expression vector. The sequence of the constructed vector was validated by sequencing. For transient transfection, the plasmids were mixed with transfection reagents at an optimal ratio and then added into the flask or the bioreactor containing HEK293. The cells were cultured in a serum-free medium and maintained in Erlenmeyer flasks on an orbital shaker or the bioreactor using a suitable stirring speed at 37 °C for 6 days. Afterwards, the cell culture broth was centrifuged, and the cell culture supernatant was loaded onto an affinity purification column at an appropriate flow rate. Finally, the protein purified by immobilized metal affinity chromatography was analyzed by SDS-PAGE.

### 2.4. Conjugation of Peptide to the Carrier Protein (KLH) for Use as an Immunogen

The designed peptides were conjugated to keyhole limpet hemocyanin (KLH) via a cysteine link. Briefly, to obtain oriented attachment of the peptide on the surface of the KLH protein, the peptides were synthetized as cysteinylated peptides with an extra N-terminally located cysteine residue. The bifunctional crosslinker GMBS (N-γ-maleimidobutyryl-oxysuccinimide ester) was used to couple peptides to the KLH carrier. The conjugation reaction was prepared by dissolving 20 mg of KLH (Calbiochem; Merck KGaA, Darmstadt, Germany) in conjugation buffer (PBS with 0.9 M NaCl, 10 mM EDTA) to a concentration of 10 mg/mL under gentle stirring for 10 min. Then, we dissolved 2 mg of active bifunctional cross-linker GMBS in 50 μL of anhydrous dimethylformamide and added 2 mL of KLH solution, mixing for 1 h at room temperature to prepare maleimide-activated KLH. Afterwards, 5 mL of HiTrap Desalting column (GE Healthcare, Chicago, IL, USA) equilibrated in conjugation buffer was used to remove the unreacted GMBS. The peptides (*w*/*w*, 20 mg of peptide) were conjugated to maleimide-activated KLH at a 1:1 ratio for 2 h at room temperature (25 °C). We dialyzed the resulting conjugates against a 100-fold excess of PBS, while the dialysis buffer was changed four times to remove the unconjugated peptide. The conjugates were centrifuged at 21,000× *g* for 15 min at 2 °C after the dialysis was finished. We confirmed the conjugation completeness, using LC–MS/MS, as the absence of free peptide in the dialysis buffer. Finally, we aliquoted the conjugates and stored them at −20 °C until the next use.

### 2.5. Preparation of Hybridomas Producing Monoclonal Antibodies Specific to Rabbit CD34 Protein

Six-week-old BALB/c mice (Charles River Laboratories, delivered by Velaz s.r.o., Prague, Czech Republic) were used to prepare hybridoma cell lines producing monoclonal antibodies specific to rabbit CD34 protein. Briefly, the mice were immunized subcutaneously with either 30 μg of synthetic peptide conjugated with KLH carrier protein (peptide 1: Ac-CNGTTTTISETRVNLTSSPGTTPSPG-NH2 or peptide 2: Ac-CDKGEELTQVLCGKEQADSEAGAR-NH2) or with 30 μg of recombinant rabbit CD34 protein in the complete Freund’s adjuvant (Merck KGaA, Darmstadt, Germany) and boosted three times at three-week intervals with 30 μg of the same antigen in the incomplete Freund’s adjuvant (Merck KGaA, Darmstadt, Germany). Then, the mice were injected intraperitoneally with 30 μg of the same antigens in PBS, and the spleens were extracted three days later. Cells from the spleens of immunized mice were fused with NS0 myeloma cells (ECACC 85110503), according to the method described by Kontsekova et al. [31]. NS0 myeloma cells were mixed with splenocytes (in a ratio 1:5) and fused for 1 min in 1 mL of 50% polyethylene glycol (PEG) 1550 (SERVA Electrophoresis GmbH, Heidelberg, Germany) in serum free Dulbecco’s modified Eagle’s medium (DMEM; ThermoFisher Scientific, Waltham, MA, USA) supplemented with 10% dimethyl sulfoxide (DMSO). The fused cells were resuspended in DMEM containing 20% horse serum, L-glutamine (2 mM), HAT media supplement Hybri-Max™, and gentamycin (40 U/mL) (both from Merck KGaA, Darmstadt, Germany) at a density of 2.5 × 10^5^ spleen cells per well in 96-well plates and incubated for 10 days at 37 °C.

The hybridoma cell lines and corresponding monoclonal antibodies specific to rabbit CD34 antigen are the exclusive property of the authors employed by NPPC-RIAP Nitra, protected by utility models (no. PUV 50129-2024 and no. PUV 50130-2024). Selected hybridoma cell lines are cryopreserved for long-term storage at NPPC-RIAP Nitra.

### 2.6. ELISA for Screening of Monoclonal Antibodies

The monoclonal antibodies specific to the CD34-derived peptides or recCD34 produced by growing hybridomas were screened using an enzyme-linked immunosorbent assay (ELISA). One of the synthetic peptides or the recCD34 protein (2 μg/mL, 50 μL/well) was coated on microtiter plates overnight at 37 °C in PBS. The coated plates were blocked with PBS-0.1% Tween 20 to reduce nonspecific binding for 1 h. Afterwards, the plates were washed with PBS-0.05% Tween 20 and incubated with 50 μL/well of hybridoma culture supernatant for 1 h at 37 °C. Goat anti-mouse immunoglobulin (Ig) conjugated with horseradish peroxidase (Dako, Glostrup, Denmark) was used to detect bound monoclonal antibodies. The reaction was developed with TMB one (Kementec Solutions A/S, Taastrup, Denmark) as a peroxidase substrate and stopped with 50 μL of 0.25 M H_2_SO_4_. The absorbance at 450 nm was measured using a PowerWave HT device (Bio-Tek; Agilent Technologies, Santa Clara, CA, USA). Readouts with an absorbance value of at least twice the value of the negative controls (PBS) were considered positive. ELISA positive hybridoma cultures were further subcloned in soft agar, according to the procedure described previously [32]. The isotype of antibodies obtained from selected hybridoma clones were determined by ELISA using a mouse Ig isotyping kit (ISO-2; Merck KGaA, Darmstadt, Germany). For the verification of the binding activity of the purified antibodies by ELISA, a three-fold serial dilution of the respective antibodies was used. A diluted mouse serum was used as the positive control, whereas PBS was applied as the negative control.

### 2.7. Flow Cytometry Analysis of mAb Activity and Specificity

Several hybridoma culture supernatants, each containing unique mAb subclones specific to peptide 1 (mouse IgG1 isotype: 58/47, 58/47/26, 58/47/34, 58/70, 139/1, 139/1/53, 139/1/59, 139/6, 264/145, 264/145/103, 264/145/117, 264/164, 320/87, 320/91, 320/91/73, 320/91/84, 433/108, 433/108/1, 433/108/12, 433/121), peptide 2 (mouse IgG1 isotype: 12/1, 12/19, 12/47, 12/47/2, 12/47/5, 16/21, 16/25, 16/25/13, 16/25/18, 16/94, 16/94/85, 16/94/90, 16/97, 16/97/11, 16/97/30, 114/3, 114/3/11, 114/3/12, 114/11, 114/11/68, 114/11/72, 114/14, 114/14/45, 114/14/55, 204/39, 204/39/19, 204/39/20, 204/42, 204/42/62, 204/42/64 a 204/43), or recCD34 (mouse IgG1 isotype: 86/55/23, 86/99/43, 86/99/49, 182/4/61, 182/7/80, 182/7/84, 533/68/34, 533/68/17, 533/73/56, 533/73/53, 533/73/89, 575/36/8, 575/36/20, 575/22/33, 575/9/79, 575/9/82 and mouse IgG2b: 715/10 and 715/16), as confirmed by ELISA screening, were selected for further flow-cytometric analyses.

The activity of selected mAb subclones was verified by incubation with special beads, which are reactive to all isotypes of mouse, rat, hamster, and rabbit antibodies (AbC™ Total Antibody Compensation Bead Kit, Thermo Fisher Scientific, Waltham, MA, USA), according to the manufacturer’s instructions. Briefly, one drop of capture beads (Component A) was incubated with 100 μL of hybridoma culture supernatants (mAb concentration at 10–50 μg/mL) for 15 min on ice and protected from light. After washing in PBS (without Ca and Mg; Biowest, Bradenton, FL USA), the beads pellets were incubated with polyclonal goat anti-mouse IgG-FITC conjugated secondary antibody (STAR117F, Bio-Rad Laboratories, Hercules, CA, USA) for 15 min on ice and protected from light. After the second wash, the beads were resuspended in PBS and immediately analyzed by flow cytometry.

The specific binding of selected mAb subclones was assessed by incubation with freshly isolated PBMCs and BMMCs (at least three samples from each mononuclear cell type). Briefly, one million cells were incubated with each hybridoma culture supernatant and goat anti-mouse IgG-FITC conjugated secondary antibody, as stated above, for capture beads staining. After the final wash in PBS, the cells were stained with dead cell marker 7-AAD (Thermo Fisher Scientific, Waltham, MA, USA) to exclude the dead cells from the flow-cytometric analysis.

At least six of the most promising mAb subclones from each group of antibodies (specific against peptide 1, peptide 2, or recCD34), in terms of the proportion of CD34^+^ cells detected within the PBMCs and BMMCs samples analyzed as described above, were selected to analyze the primitive hematopoietic cell populations (CD34^+^CD45^−^ and CD34^+^CD45^+^) in rabbit peripheral blood or bone marrow by flow cytometry. Briefly, one million PBMCs or BMMCs were double-stained with the specific IgG1 or IgG2b subclone of CD34 mAb (hybridoma culture supernatants) and the specific anti-CD45 antibody (mouse IgG2a, clone ISC18A; WSU, Pullman, WA, USA) for 15 min on ice and protected from light. After washing, the cells were incubated for 15 min on ice with either anti-mouse IgG1-FITC, anti-mouse IgG2b-FITC, or anti-mouse IgG1-APC conjugated secondary antibody, for CD34 mAb subclones, and anti-mouse IgG2a-PE conjugated secondary antibody for CD45 primary antibody. After the final wash, the samples were stained with 7-AAD to exclude dead cells from the analysis.

The samples were analyzed immediately after the staining and/or washing procedure using a FACSCalibur flow cytometer (BD Biosciences, San Jose, CA, USA) equipped with a 488 nm argon ion laser and a red-diode (635 nm) laser. Fluorescent signals were acquired by Cell Quest Pro ™ software version 6.0 (BD Biosciences, San Jose, CA, USA) in a green FL1 channel using a 530/30 nm band pass filter, an orange FL2 channel using a 585/42 nm band pass filter, a red FL3 channel using a 670 nm long pass filter, and/or a far-red FL4 channel using a 661/16 nm band pass filter. Calibration of the instrument was performed periodically using standard calibration beads (BD CaliBRITE™; BD Biosciences, San Jose, CA, USA). At least 10,000 or 25,000 events (capture beads and mononuclear cells, respectively) were acquired for each sample. Unstained samples (beads or cells) or samples stained with secondary antibodies were used as control samples to gate the positive cells, according to the increased fluorescent intensity. The obtained flow-cytometric data were evaluated using FlowJo™ v10.10.0 Software (BD Biosciences, San Jose, CA, USA).

### 2.8. Western Blot Analysis of mAb Specificity

To confirm the specific binding of the produced mAbs to CD34 protein in the rabbit biological samples, Western blot analysis was performed. Briefly, PBMC and BMMC samples (3–5 × 10^6^) were lysed in 200 µL of 1× SDS sample buffer (pH 6.8; 50 mM Tris-HCl, 2% SDS, 200 mM 2-mercapto ethanol, 0.03% bromophenol blue, 10% glycerol). Rabbit mesenchymal stem cells (MSCs) from bone marrow and endothelial progenitor cells (EPCs) from peripheral blood, isolated as described previously [27,33], were also lysed as mentioned above and used as negative controls. Recombinant rabbit CD34 (recCD34) protein was used as positive control. Rabbit cell samples and recCD34 protein were boiled for 5 min prior to being subjected to SDS-PAGE (90 V; 2 h) in a 4–15% gradient gel, together with a molecular ladder (Precision Plus Protein WesternC Standards; Bio-Rad Laboratories, Hercules, CA, USA). Moreover, unboiled recCD34 protein was included as an additional positive control. The proteins were transferred from gel to an 0.2 µm PVDF membrane using TransBlot Turbo (Bio-Rad Laboratories, Hercules, CA, USA) for 7 min at 25 V and 2.5 A. The membrane was blocked in 5% non-fat dry milk in TBS-0.1% Tween 20 buffer for 2 h and incubated overnight at 4 °C with selected mAb subclones (hybridoma culture supernatants diluted at ratio 1:10) specific against peptide 1, peptide 2, or recCD34. The expression of vinculin as the internal reference protein was detected in all cell samples using a specific mouse antibody (clone VIIF9; Merck KGaA, Darmstadt, Germany). After washing in 1% non-fat dry milk in TBS-0.2% Tween 20, the membrane was incubated with HRP-conjugated sheep anti-mouse IgG antibody (1:5000; Amersham, UK) for 1 h at room temperature. Then, the membrane was washed and incubated in ECL substrate for 5 min and visualized using the ChemiDoc system (Bio-Rad Laboratories, Hercules, CA, USA).

### 2.9. Purification of Selected mAbs Specific to Rabbit CD34 and Their Quality Control

We chose two specific mAbs from each group of positively tested hybridoma culture supernatants (58/47/26 and 58/47/34 against peptide 1, and mAb 182/7/80 and 575/36/8 against recCD34) for further purification. Briefly, serum-free hybridoma culture supernatant was adjusted to pH 8.0 (1 M Tris-HCl pH 9.0), followed by centrifugation (20,000× *g* at 4 °C) and filtration (0.2 µm filter). Purification of the antibodies was performed by affinity chromatography on a Protein G HP column (GE Healthcare, Chicago, IL, USA). The eluted antibody fractions were pooled, and the pH was adjusted to 7.5–8.0. In the next step, the antibody was purified using a HiTrap Q HP 5 mL column. The collected fractions were desalted using a HiPrep Desalting column (GE Healthcare, Chicago, IL, USA) in PBS. The purified antibody was concentrated via ultrafiltration using an Amicon Ultra-15 (Merck KGaA, Darmstadt, Germany) and stored in PBS in working aliquots at −70 °C. The antibody concentration was determined using a Bio-Rad Protein assay (Bio-Rad Laboratories, Hercules, CA, USA). The purity of the antibody was subsequently verified by electrophoresis (SDS-PAGE, as described above) and Coomassie blue staining. The activity of the purified antibodies was verified by the ELISA three-fold serial dilution of the respective antibodies, as described above, starting at a concentration of 450 ng/mL. In addition, the specific binding of the purified mAbs was confirmed using flow cytometry by incubation with BMMC samples and by co-staining with the CD45 antibody, as described above.

### 2.10. Statistical Analyses

The obtained data were evaluated using GraphPad Prism version 9.5.1 for Windows (GraphPad Software, San Diego, CA, USA) with one-way ANOVA, followed by Fisher’s LSD test. The results are expressed as the mean ± SD. *p*-values at *p* < 0.05 were considered as statistically significant.

## 3. Results

### 3.1. Production of recCD34 Protein

Recombinant rabbit CD34 protein, representing the extracellular domain of predicted rabbit CD34, was successfully produced and purified, with a varying molecular weight ranging from 55 to 82 kDa, which was probably due to different post-translational modifications of the produced recombinant protein in the HEK293 cells. However, a specific molecular weight of about 32 kDa was observed for the recombinant protein after the deglycosylation process, which nearly corresponds to the predicted molecular weight of the recombinant CD34 protein (29 kDa), according to the designed amino acid sequence of the extracellular domain.

### 3.2. ELISA Screening of mAbs Specific to Rabbit CD34 Immunogen

**Figure 3:**
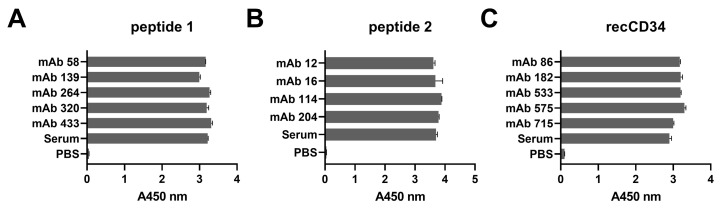
Immunoreactivity of selected monoclonal antibodies in ELISA. mAbs from undiluted hybridoma clone culture supernatants generated against peptide 1 (**A**), peptide 2 (**B**), or recCD34 (**C**). Serum diluted at the ratio of 1:1000 in PBS was used as the positive control. Results are expressed as mean ± SD.

We immunized mice with either the synthetic peptide (1 and 2) derived from rabbit CD34 or with recombinant rabbit CD34 protein to prepare antibodies with specificity to rabbit CD34 antigen. Screening of the antibody-expressing hybridoma clones generated from the peptide 1-immunized mice revealed that five monoclonal antibodies targeted peptide 1, as documented in Figure 3A. Next, we obtained four monoclonal antibodies with specific immunoreactivity to peptide 2 after the immunization of mice with peptide 2 (Figure 3B). With the aim of preparing monoclonal antibodies with the ability to detect whole rabbit CD34 protein, recombinant protein was used as the immunogen. Screening revealed five positive hybridoma clones producing antibodies which recognize recombinant rabbit CD34 protein (Figure 3C).

### 3.3. Flow Cytometry

**Figure 4:**
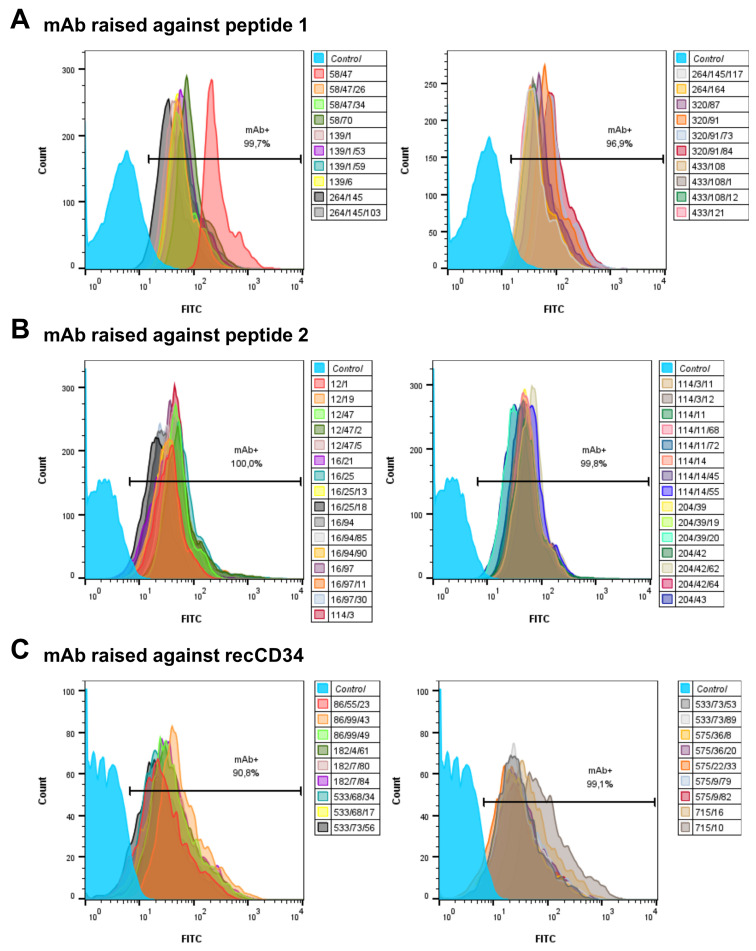
Flow-cytometric staining of capture beads with different hybridoma supernatants. Binding activity of mAbs from hybridoma subclone supernatants assessed by flow-cytometric analysis using incubation of capture beads with respective antibodies generated against peptide 1 (**A**), peptide 2, (**B**) or recCD34 (**C**).

The tested mouse mAb subclones from each group of antibodies (produced against peptide 1, peptide 2, and recCD34) showed high antibody activity, since all of them strongly bound to the capture beads (>90% positivity; Figure 4).

**Figure 5:**
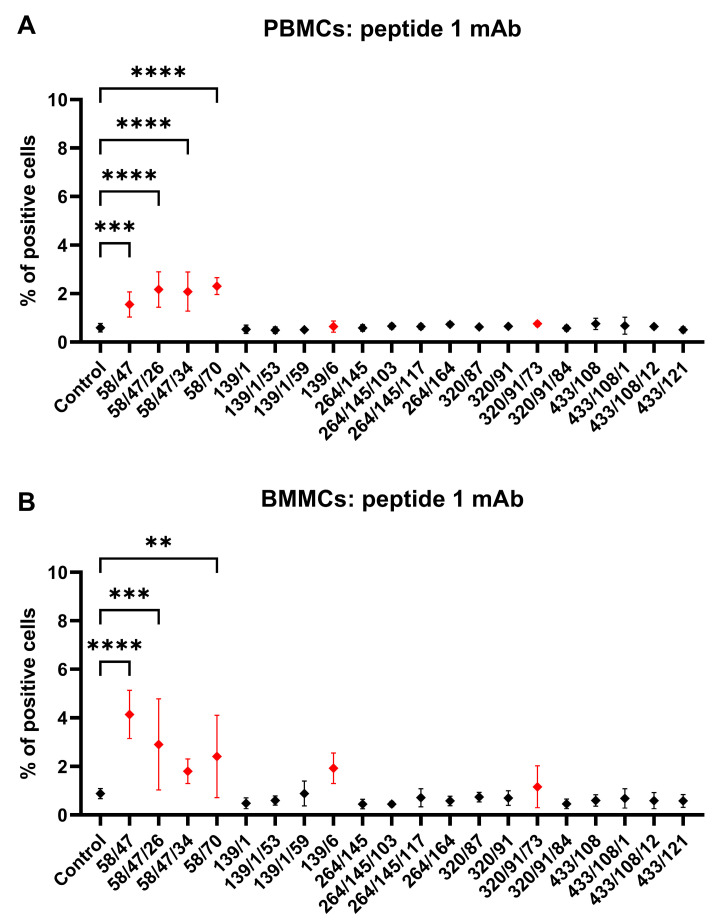
Flow-cytometric staining of blood and bone marrow cells with hybridoma supernatants (peptide 1). Reactivity of mAbs from hybridoma subclone supernatants generated against peptide 1 with CD34 antigen in PBMC (**A**) and BMMC (**B**) samples. Six mAbs selected for subsequent detection of CD34^+^CD45^−^ and CD34^+^CD45^+^ cell subsets within PBMC and BMMC samples are shown in red. Results are expressed as mean ± SD. Difference is statistically significant at ** *p* < 0.01, *** *p* < 0.001, and **** *p* < 0.0001.

**Figure 6:**
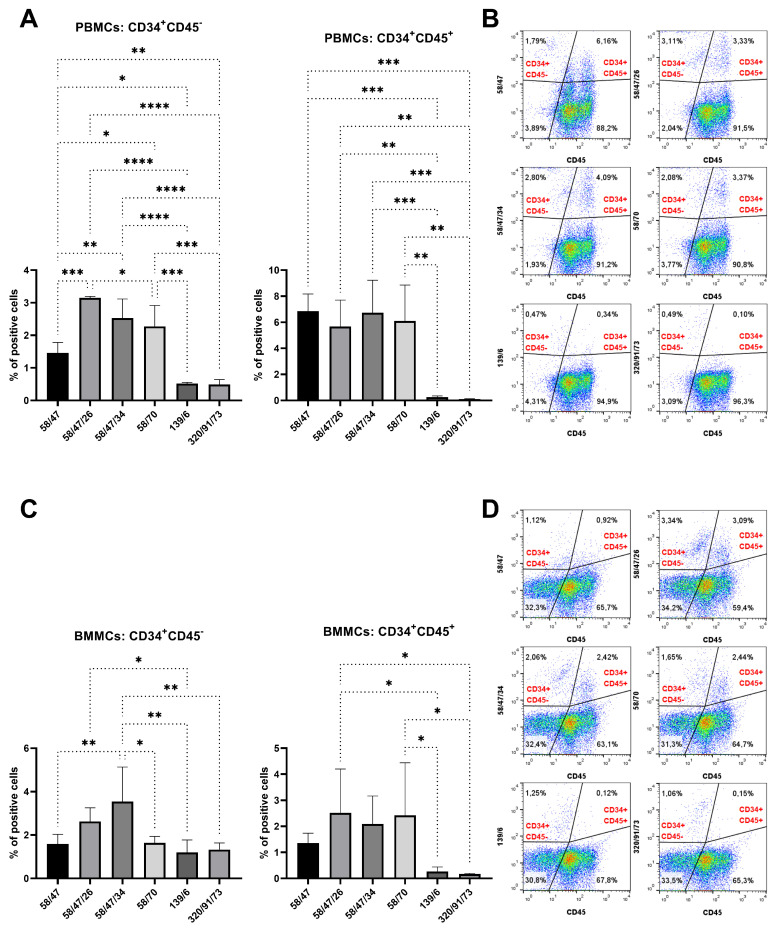
Co-staining of blood and bone marrow cells with six selected hybridoma supernatants (peptide 1) and CD45 antibody. Detection of CD34^+^CD45^−^ and CD34^+^CD45^+^ cell subsets within PBMC (**A**) and BMMC (**C**) samples as shown in illustrative flow-cytometric dot plots for blood and bone marrow ((**B**) and (**D**), respectively). Results are expressed as mean ± SD. Difference is statistically significant at * *p* < 0.05, ** *p* < 0.01, *** *p* < 0.001, and **** *p* < 0.0001.

We incubated rabbit PBMCs and BMMCs with mAb subclones developed against peptide 1 to analyze their specific binding to CD34. Four of these mAbs (58/47, 58/47/26, 58/47/34, and 58/70) detected a significantly (*p* < 0.001) positive subset of CD34^+^ cells (about 2%) within the PBMC samples compared to the results for the control samples (Figure 5A). Three of these subclones (58/47, 58/47/26, and 58/70) also detected positive CD34^+^ cells (2–4%) within the BMMC samples compared to the results for the control samples (*p* < 0.01, Figure 5B). Moreover, subclones 58/47/34, 139/6, and 320/91/73 detected higher populations of CD34^+^ within the BMMCs compared to the results for the control sample, although the levels were not significant. We selected six subclones (58/47, 58/47/26, 58/47/34, 139/6, and 320/91/73) for the subsequent detection of CD34^+^CD45^−^ and CD34^+^CD45^+^ cell subsets within the PBMC and BMMC samples. A significantly (*p* < 0.05) higher number of CD34^+^CD45^−^ PBMCs (1–3%) was observed using subclones 58/47, 58/47/26, 58/47/34, and 58/70 in comparison with the results for the 139/6 and 320/91/73 subclones. Similarly, a significantly (*p* < 0.01) higher number of CD34^+^CD45^+^ PBMCs (about 6%) was observed using subclones 58/47, 58/47/26, 58/47/34, and 58/70 in comparison with the results for the 139/6 and 320/91/73 subclones (Figure 6A). In the bone marrow, we observed up to 3.5% of CD34^+^CD45^−^ BMMCs stained by mAb 58/47/34, which was significantly (*p* < 0.05) more than the results obtained by staining with the other five subclones. About 2.5% of CD34^+^CD45^+^ cells were observed in BMMCs using the 58/47/26 and 58/70 subclones, which was significantly higher (*p* < 0.05) in comparison to the results for the subclones 139/6 and 320/91/73, but not significant compared to the results for the subclones 58/47 and 58/47/34 (Figure 6C).

**Figure 7:**
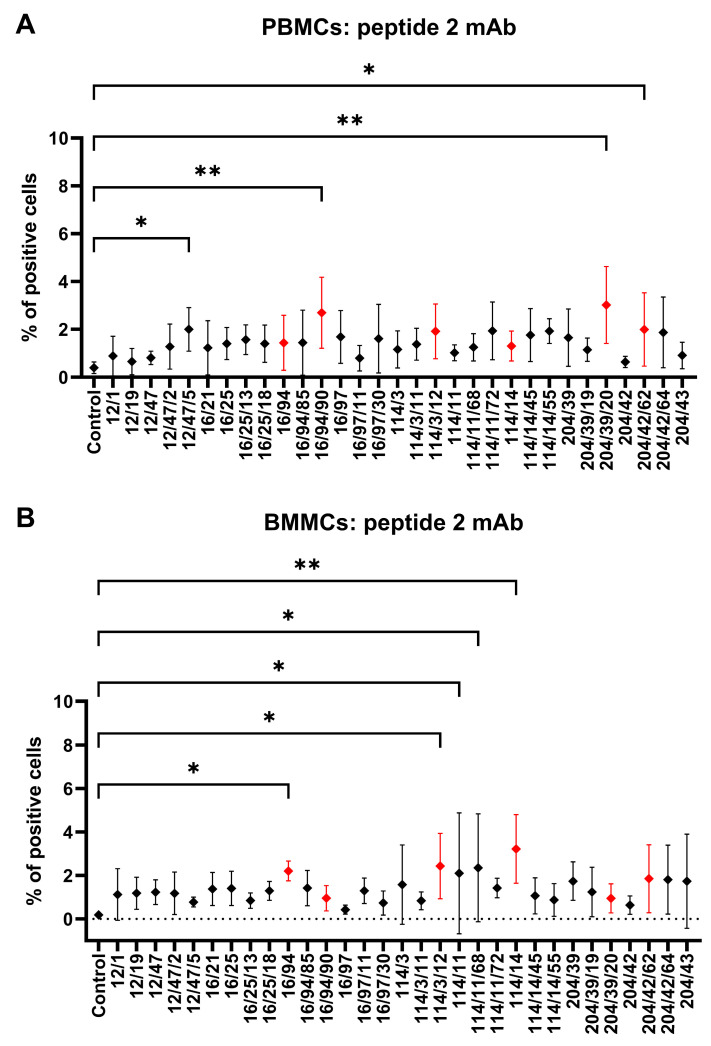
Flow-cytometric staining of blood and bone marrow cells with hybridoma supernatants (peptide 2). Reactivity of mAbs from hybridoma subclone supernatants generated against peptide 2 with CD34 antigen in PBMC (**A**) and BMMC (**B**) samples. Six mAbs selected for subsequent detection of CD34^+^CD45^−^ and CD34^+^CD45^+^ cell subsets within PBMC and BMMC samples are shown in red. Results are expressed as mean ± SD. Difference is statistically significant at * *p* < 0.05 and ** *p* < 0.01.

**Figure 8:**
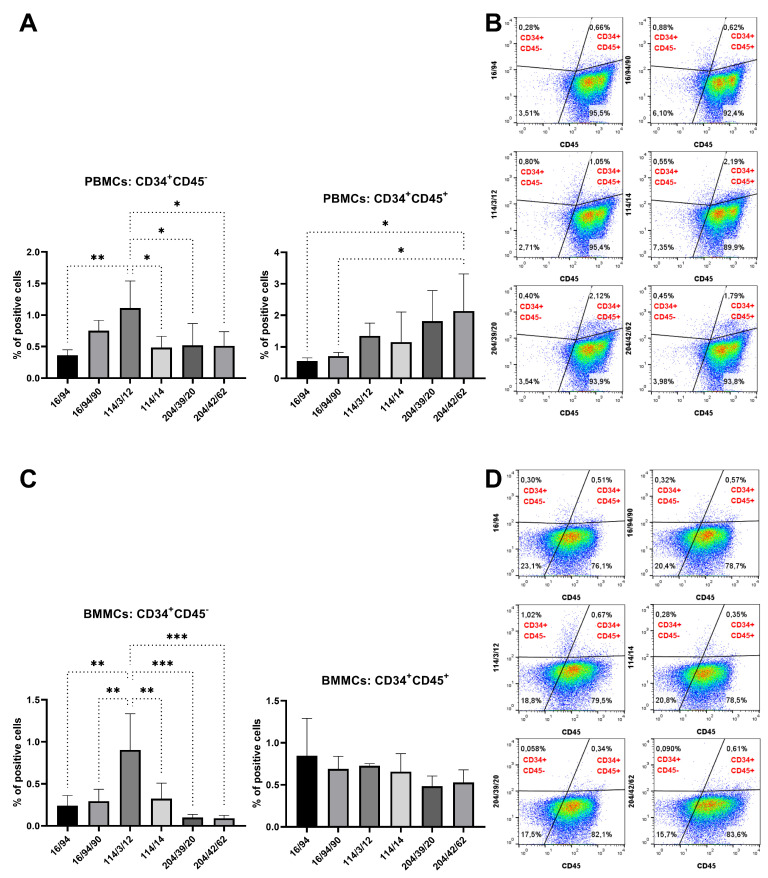
Co-staining of blood and bone marrow cells with six selected hybridoma supernatants (peptide 2) and CD45 antibody. Detection of CD34^+^CD45^−^ and CD34^+^CD45^+^ cell subsets within PBMC (**A**) and BMMC (**C**) samples as shown in illustrative flow-cytometric dot plots for blood and bone marrow ((**B**) and (**D**), respectively). Results are expressed as mean ± SD. Difference is statistically significant at * *p* < 0.05, ** *p* < 0.01, and *** *p* < 0.001.

Rabbit PBMCs and BMMCs were incubated with mAb subclones developed against peptide 2 to analyze their specific binding to CD34. Four of them (12/47/5, 16/94/90, 204/39/20, and 204/42/62) revealed a significantly (*p* < 0.05) positive subset of CD34^+^ cells (2–3%) within PBMC samples compared to the results for the control samples (Figure 7A). Another four subclones (16/94, 114/3/12, 114/11, and 141/14) similarly detected 2–3% of positive CD34^+^ cells within the BMMC samples compared to the results for the control samples (*p* < 0.05, Figure 7B). Six selected subclones (16/94, 16/94/90, 114/3/12, 141/14, 204/39/20, and 204/42/62) were used for detecting of CD34^+^CD45^−^ and CD34^+^CD45^+^ cell subsets within the PBMC and BMMC samples. The highest (*p* < 0.05) number of CD34^+^CD45^−^ PBMCs (1%) was observed by the subclone 114/3/12 in comparison to the results for the other subclones. On the other hand, the highest (*p* < 0.05) number of CD34^+^CD45^+^ PBMCs (2%) was observed by the subclone 204/42/62 compared to the results for the other subclones (Figure 8A). We noticed up to 1% of CD34^+^CD45^−^ BMMCs stained by mAb 114/3/12, which was significantly (*p* < 0.01) more than those resulting from staining with other subclones. About 0.5–1% of CD34^+^CD45^+^ cells were observed in BMMCs using all subclones, without statistically significant differences (Figure 8C).

**Figure 9:**
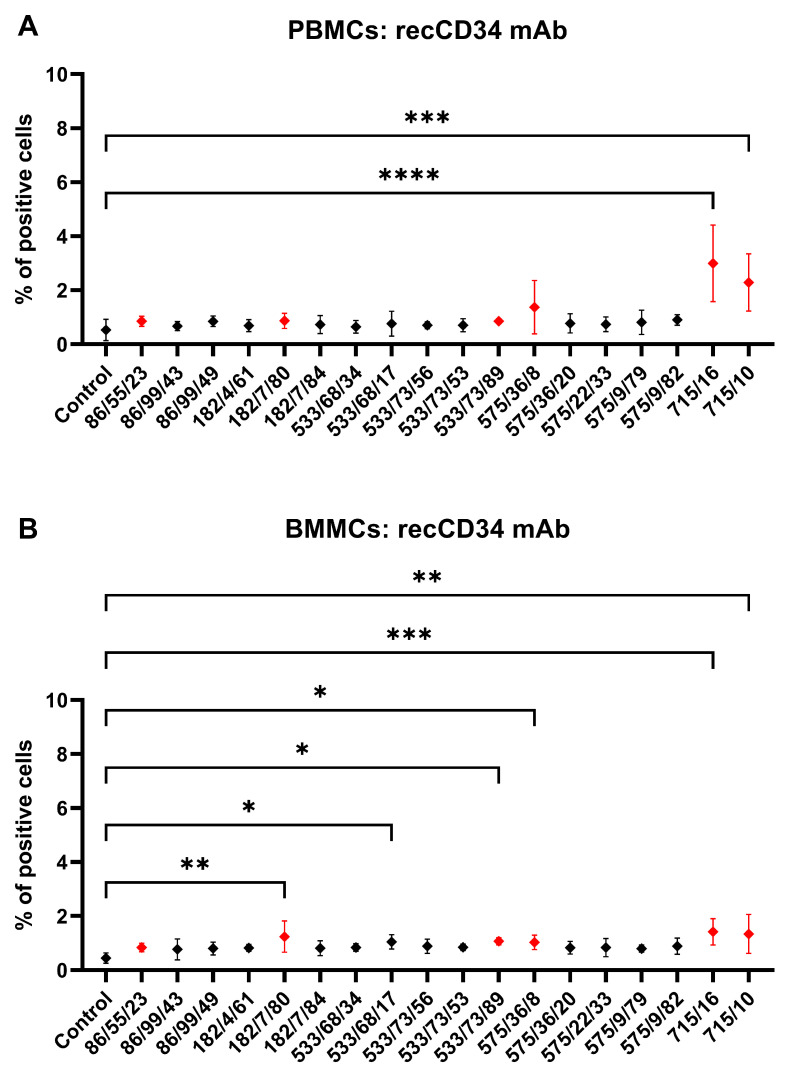
Flow-cytometric staining of blood and bone marrow cells with hybridoma supernatants (recCD34). Reactivity of mAbs from hybridoma subclone supernatants generated against recCD34 with CD34 antigen in PBMC (**A**) and BMMC (**B**) samples. Six mAbs selected for subsequent detecting of CD34^+^CD45^−^ and CD34^+^CD45^+^ cell subsets within PBMC and BMMC samples are shown in red. Results are expressed as mean ± SD. Difference is statistically significant at * *p* < 0.05, ** *p* < 0.01, *** *p* < 0.001, and **** *p* < 0.0001.

**Figure 10:**
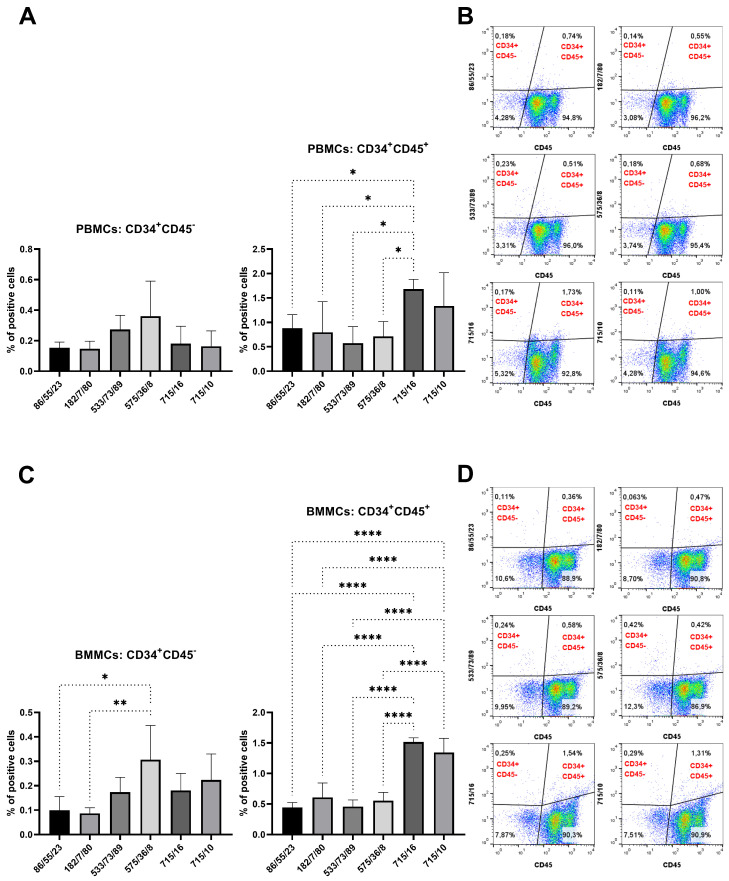
Co-staining of blood and bone marrow cells with six selected hybridoma supernatants (recCD34) and CD45 antibody. Detection of CD34^+^CD45^−^ and CD34^+^CD45^+^ cell subsets within PBMC (**A**) and BMMC (**C**) samples as shown in illustrative flow-cytometric dot plots for blood and bone marrow ((**B**) and (**D**), respectively). Results are expressed as mean ± SD. Difference is statistically significant at * *p* < 0.05, ** *p* < 0.01, and **** *p* < 0.0001.

Finally, PBMCs and BMMCs were incubated with mAb subclones developed against recCD34 to analyze their specific binding to CD34. Two of them (715/16 and 715/10) revealed a significantly (*p* < 0.001) positive subset of CD34^+^ cells (2–3%) within the PBMC samples compared to the results for the control samples (Figure 9A). Moreover, the same subclones, together with subclones 182/7/80, 533/68/17, 533/73/89, and 575/36/8, revealed up to 1.5% positive CD34^+^ cells within the BMMC samples compared to the results for the control samples (*p* < 0.05, Figure 9B). Six subclones (182/7/80, 533/68/17, 533/73/89, 575/36/8, 715/16, and 715/10) were selected to detect CD34^+^CD45^−^ and CD34^+^CD45^+^ cell subsets within the PBMC and BMMC samples. No significant differences were found in the number of CD34^+^CD45^−^ PBMCs (<0.5%). On the other hand, subclone 715/16 detected the highest (*p* < 0.05) number of CD34^+^CD45^+^ PBMCs (1.5%) compared to the results for the other subclones, except for subclone 715/10 (Figure 10A). We observed up to 0.3% of CD34^+^CD45^−^ BMMCs stained by mAb 575/36/8, which was significantly (*p* < 0.05) more than the number obtained by staining with other subclones. About 1.5% of CD34^+^CD45^+^ cells were observed in BMMCs using subclones 715/16 and 715/10, significantly higher (*p* < 0.05) in comparison to results for the other subclones (Figure 10C).

### 3.4. Western Blot

**Figure 11:**
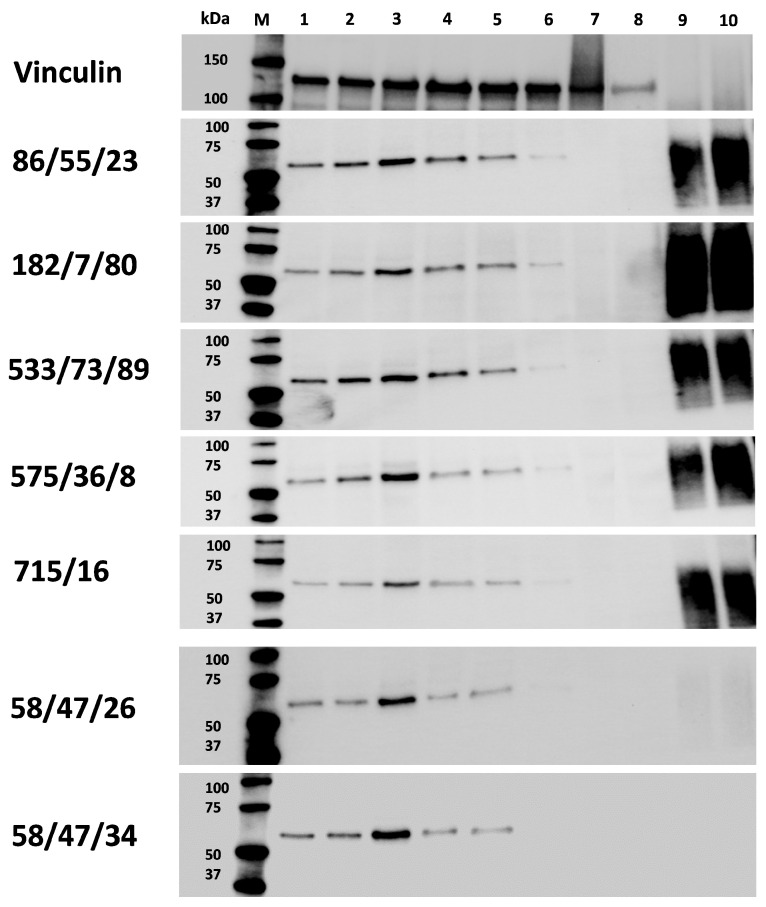
Detection of rabbit CD34 protein in denaturated samples. Western blot analysis of selected mAbs from hybridoma subclone supernatants generated against recCD34 and peptide 1. M—ladder; BMMC samples (1, 2, 3); PBMC samples (4, 5, 6); MSCs sample (7); EPC sample (8); unboiled recCD34 protein (9); boiled recCD34 protein (10). Western blot original images can be found in Figures S1–S8.

At first, we compared the ability of selected mAbs generated against peptide 1 (58/47, 58/47/26, 58/47/34) and peptide 2 (16/94, 114/3/12, 204/42) to detect target CD34 protein in PBMC and BMMC samples. All mAbs against peptide 1 bound to a specific peptide on the PVDF membrane, whereas no specific binding was observed for mAbs produced against peptide 2. Then, selected mAbs against peptide 1 (58/47/26 and 58/47/34) and mAbs against recCD34 (86/55/23, 182/7/80, 533/73/89, 575/36/8, and 715/16) were compared for their specific binding with rabbit CD34 protein in different biological samples and their specific binding with the recCD34 protein itself. The Western blot analysis confirmed the specificity of all tested mAb subclones (against peptide 1 as well as against recCD34), as a specific protein with a size ranging between 50–75 kDa was detected in all BMMC samples. A slightly less intensive signal of the specific protein was also observed in all PBMC samples. The size of the CD34 protein detected in the BMMC and PBMC samples corresponded with those of both positive controls (unboiled and boiled recCD34). In rabbit MSCs and EPCs, no specific protein of the target size was observed (Figure 11). Interestingly, mAbs produced against peptide 1 (58/47/26 and 58/47/34, respectively) showed very slight or no detection of recCD34 protein. Probably, the epitope recognized by the peptide 1 monoclonal antibodies is scarcely accessible in recCD34 protein.

### 3.5. Quality of Purified mAbs Specific to Rabbit CD34

**Figure 12:**
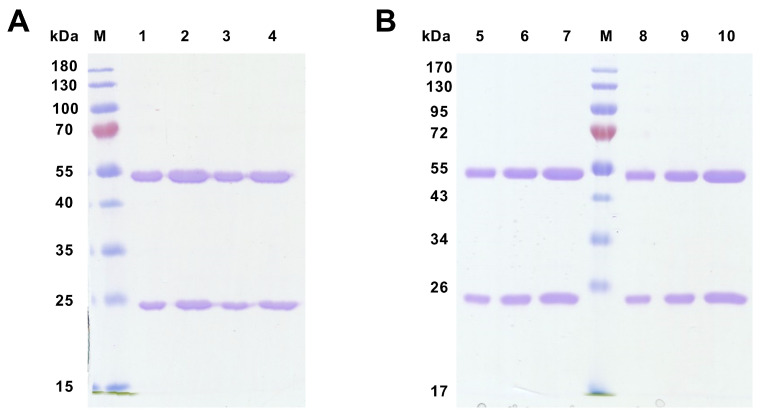
Quality control of purified monoclonal antibodies using SDS-PAGE. Coomassie blue-stained 12% SDS-PAGE gel shows the quality of purified mAbs generated against peptide 1 (**A**) and against recCD34 protein (**B**). M—prestained ladder; 1—mAb 58/47/26 (1 µg); 2—mAb 58/47/26 (1 µg); 3—mAb 58/47/34 (1 µg); 4—mAb 58/47/34 (2 µg); 5—mAb 182/7/80 (0.5 µg); 6—mAb 182/7/80 (1 µg); 7—mAb 182/7/80 (2 µg); 8—mAb 575/36/8 (0.5 µg); 9—mAb 575/36/8 (1 µg); 10—mAb 575/36/8 (2 µg).

Supernatants from selected hybridoma clones were used for the purification process of specific mAb. Purified mAb against peptide 1 (58/47/26 and 58/47/34) and against recCD34 (182/7/80 and 575/36/8) were analyzed in terms of their quality via SDS-PAGE electrophoresis, ELISA, and flow cytometry. Due to electrophoresis, reduced samples of purified mAbs were successfully separated into heavy (50 kDa) and light (25 kDa) chains (Figure 12), confirming their purity.

**Figure 13:**
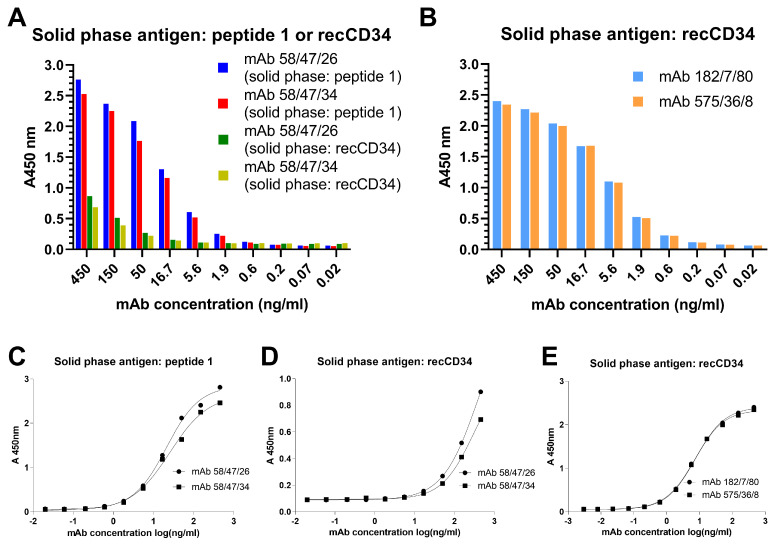
ELISA immunoreactivity of purified monoclonal antibodies. Binding activity of purified mAbs assessed by ELISA three-fold serial dilution of respective antibodies generated against peptide 1 (**A**) or recCD34 (**B**). Peptide 1 and recCD34 protein were used as solid phase antigens at a concentration of 2.5 μg/mL for mAb 58/47/26 and 58/47/34 (**A**). recCD34 protein was used as solid phase antigen at a concentration of 2 μg/mL for mAb 182/7/80 and 575/36/8 (**B**). Results are expressed as mean values. Determination of EC50 values for mAb 58/47/26 and 58/47/34 against peptide 1 (**C**) or recCD34 (**D**) as solid phase and for mAb 182/7/80 and 575/36/8 against recCD34 (**E**) as solid phase, calculated using GraphPad Prism software version 9.5.1 for Windows fitting to a four-parameter logistic curve.

The immunoreactivity of the purified mAbs was verified by ELISA assay using wells precoated with peptide 1 and/or recCD34 (Figure 13). Concentrations of purified mAbs at 450 and 150 ng/mL exhibited a nearly two-fold higher immunoreactivity of mAb 58/47/26 and 58/47/34 for peptide 1 compared to the results for recCD34 (Figure 13A). This finding corresponds to the slight reactivity of these mAbs with recCD34 revealed by Western blot analysis. Moreover, the dilution of these mAbs more rapidly decreased the immunoreactivity of mAb 58/47/26 and 58/47/34 for recCD34 compared to that of peptide 1. On the other hand, the immunoreactivity of mAb 182/7/80 and 575/36/8 to recCD34 was nearly 1,5-fold higher (Figure 13B) than the immunoreactivity of mAb 58/47/26 and 58/47/34 to recCD34 (Figure 13A) at the concentrations of 450 and 150 ng/mL. Moreover, the diluted mAb 182/7/80 and 575/36/8 still exhibited positivity at the concentration of 5.6 ng/mL (Figure 13B). Determination of EC50 values confirmed the strong binding of mAb 58/47/26 and 58/47/34 to peptide 1 (20.9 and 25.4 ng/mL, respectively; Figure 13C), while high EC50 values of these mAbs displayed weak binding to recCD34 (392.3 and 286.6 ng/mL, respectively; Figure 13D). On the contrary, the EC50 values of mAb 182/7/80 and 575/36/8 showed strong binding to recCD34 (7.5 and 7.1 ng/mL, respectively; Figure 13E).

**Figure 14:**
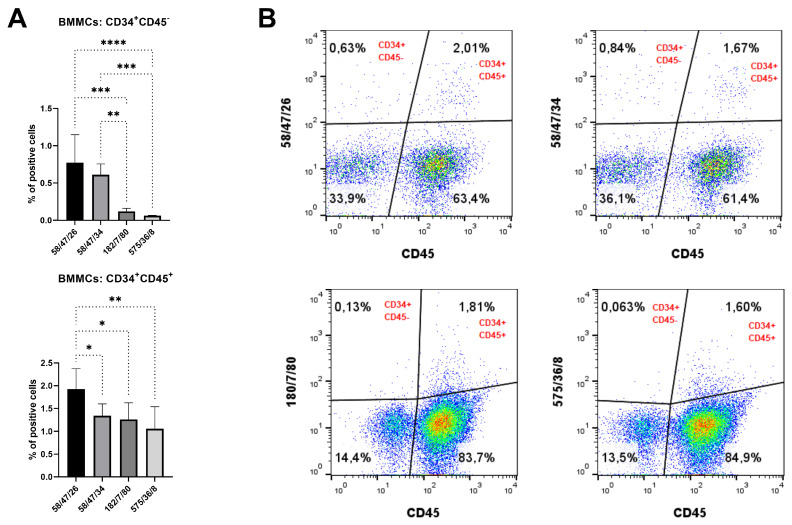
Flow-cytometric staining of bone marrow cells with purified monoclonal antibodies. Binding activity of purified mAb generated against peptide 1 (mAb 58/47/26 and 58/47/34) or recCD34 (mAb 182/7/80 and 575/36/8), assessed by flow cytometry. Proportion of CD34^+^CD45^−^ and CD34^+^CD45^+^ cell subpopulations within BMMC samples (**A**). Results are expressed as mean ± SD. Difference is statistically significant at * *p* < 0.05, ** *p* < 0.01, *** *p* < 0.001, and **** *p* < 0.0001. Illustrative flow-cytometric dot plots of detected CD34 and CD45 positive cells in BMMC samples (**B**).

The specific binding of purified mAb to CD34 in native cell samples was assessed by flow cytometry. Purified mAb detected about 0.1–0.8% of CD34^+^CD45^−^ cells in BMMC samples. On the other hand, 1–2% of BMMCs were concurrently positive for CD34 and CD45 (Figure 14), as detected by purified mAbs.

## 4. Discussion

CD34, a cell surface protein, has been used for more than 40 years to identify and isolate hematopoietic stem and progenitor cells for the transplantation of bone marrow. In addition to their presence in hematopoietic stem cells and their progenitors, CD34 expression was also observed in human or mouse mesenchymal stem cells, muscle satellite cells, keratocytes, interstitial cells, fibrocytes, epithelial and endothelial progenitors [10,29,34]. In rabbits, we observed increased CD34 expression in CD45 depleted hematopoietic cells [35]. On the other hand, decreased CD34 expression was observed in the endothelial progenitors after several passages [27], and we did not find expression of CD34 in rabbit mesenchymal stem cells derived from bone marrow or adipose tissue [33,36]. In all these studies, the expression of CD34 was mainly analyzed at the molecular (mRNA) level due the lack of antibodies specific to rabbit CD34 protein [26].

Here, we presented novel monoclonal antibodies specific to rabbit CD34 protein developed using hybridoma technology. The same principle has been applied to produce mouse mAbs specific to canine, porcine, or ovine CD34 [15,16,17]. Moreover, a similarly produced anti-CD117 monoclonal antibody was reported to be specific for porcine hematopoietic stem cells and their progenitors [37]. On the other hand, a rabbit polyclonal antibody specific to bovine CD34 has also been developed [38]. To detect canine CD34, a CD34-murine Ig fusion recombinant protein (CD34-Ig), derived from the extracellular domain of canine CD34 protein, was used as the immunogen to firstly generate affinity-purified polyclonal antiserum (RPaCD34). Although this antiserum reacted with about 1% of canine BMMCs, it was not optimal for the sorting of CD34^+^ cells, according to the authors. Therefore, a combination of CD34-Ig protein with canine CD34^+^ leukemic cell lines was additionally injected into mice to produce canine-specific CD34 mAbs [15]. Due to the lack of rabbit CD34^+^ cell lines, we decided to design two synthetic peptides derived from the extracellular domain of predicted rabbit CD34 protein (Figure 1), which were used as immunogens. Moreover, to imitate the native structure of rabbit CD34 protein, a recombinant CD34 protein of the extracellular domain of predicted rabbit CD34 protein (Figure 2) was commercially produced in a eukaryotic expression system and applied as the other immunogen for hybridoma generation. In other study, mice were immunized with recombinant sheep CD34 plasmid to generate sheep-specific CD34 mAb [17]. In addition, recombinant proteins representing the extracellular domain of porcine or bovine CD34 protein were prepared in a prokaryotic expression system for animal immunization to obtain specific mouse anti-porcine monoclonal or rabbit anti-bovine polyclonal antibodies [16,38].

Hybridoma clones prepared in this study were screened for specific antibody production via the ELISA method using plates precoated with synthetic peptide or recCD34, depending on the immunogen used for their generation. We obtained five hybridoma clones positive for peptide 1, four clones positive for peptide 2, and at least five clones positive for recCD34 (Figure 4), which were further subcloned to produce CD34-specific mAbs. At the end of this process, we obtained 20 hybridoma subclones specific against peptide 1, 31 subclones specific against peptide 2, and 18 subclones specific against recCD34. Similarly, McSweeney et al. [15] generated 32 hybridomas reactive with CD34-Ig protein, while 21 of them also reacted with canine leukemic cell lines. As we successfully proved the high antibody affinity of produced supernatants (Figure 4), we further analyzed the binding activity of supernatants from all 69 produced hybridoma subclones via single or double staining with rabbit PBMCs and BMMCs and anti-rabbit CD45 antibody, respectively. Six mAbs from each antibody group exhibited increased reactivity with those samples, with some differences (Figure 6, Figure 8 and Figure 10). mAb 58/47, 58/47/26, 58/47/34, and 58/70 produced against peptide 1 detected 2–6% and 2–4% of positive cells in PBMCs and BMMCs, respectively. mAb 16/94, 16/94/90, 114/3/12, 141/14, 204/39/20, and 204/42/62 produced against peptide 2 reacted with 2–3% of PBMCs, as well as with BMMCs. mAb 182/7/80, 533/68/17, 533/73/89, 575/36/8, 715/16, and 715/10 detected 0.5–3% of positive PBMCs and 0.5–1.5% of positive BMMCs. Although the proportion of positive PBMCs and BMMCs varied among the used mAbs, the values of bone marrow positive cells are in accordance with about 3% of positive cells observed in canine bone marrow by the produced hybridoma supernatants [15]. In human bone marrow, the proportion of CD34^+^ cells ranges between 1% and 5% [39,40], which agrees with our findings in rabbit BMMCs. However, 4% to 17% of CD34^+^ cells were detected in mouse bone marrow [41]. These findings indicated that the proportion of bone marrow cells positive for CD34 can vary, not only within the same species but also among the species themselves. Porada et al. [17] observed about 1% of CD34^+^ cells in sheep bone marrow, about 2% of CD34^+^ in sheep peripheral blood, and about 4% of positive cells in sheep cord blood using the newly produced specific CD34 mAb. On the contrary, less than 0.1% of CD34^+^ cells were detected in canine peripheral blood [15], which is relatively low in comparison with our obtained data for rabbit blood cells or for the abovementioned ovine blood cells. In addition, about 4% of CD34^+^ cells were detected in porcine bone marrow by specifically generated CD34 mAb [16]. Moreover, these authors analyzed additional tissues for the presence of CD34^+^ cells using the new antibody (SwCD34) and flow cytometry in cord blood (10%), ovary (9%), endometrium (10%), lung (2%), liver (3%), kidney (5%) and skeletal muscle (less than 0.5%). However, we did not analyze other tissues in rabbits except for the above stated bone marrow and peripheral blood samples. 

To prove the specific binding of mAbs secreted in hybridoma supernatants produced in this study against the rabbit CD34 immunogen, we performed Western blot analysis (Figure 11). We observed the very low or unspecific binding of hybridoma subclones generated against peptide 2. On the other hand, several hybridoma subclones generated against peptide 1 (mAb 58/47/26 and 58/47/34) or against recCD34 protein (mAb 86/55/23, 182/7/80, 533/73/89, 575/36/8, and 715/16) recognized a specific band of the same size within a range of 50 to 75 kDa in all bone marrow and the majority of peripheral blood samples. Moreover, mAb 86/55/23, 182/7/80, 533/73/89, 575/36/8, and 715/16 specifically detected recCD34 protein in the same size range as was noticed for CD34 protein in rabbit blood and bone marrow samples. On the other hand, mAb 58/47/26 and 58/47/34 did not properly recognized recCD34 protein, probably due to its different binding sites or epitopes. In addition, we did not detect CD34 protein in MSC and EPC samples, which is in agreement with our previous studies, in which CD34 expression was not found at the mRNA level in such samples [27,33]. Moreover, the commercially produced recCD34 protein, which represents the extracellular domain of predicted rabbit CD34, possesses a molecular weight of about 50 to 80 kDa. Nevertheless, after the deglycosylation process, a band with a specific size of about 32 kDa was detected. This finding fully corresponded with the size of predicted rabbit CD34 protein (G1SJT2) in its full length, which is about 40 kDa [28]. We can therefore assume that the band detected within rabbit BMMCs and PBMCs is a specific CD34 protein with related posttranslational modifications, which results in a protein size between 50 and 75 kDa. In humans, the full-length sequence of CD34 cDNA predicts a protein of about 40 kDa [42]. However, the de-N-glycosylated and desialylated forms of CD34 antigen are reported to measure about 90 kDa and 150 kDa, respectively [43]. In addition, several human-specific CD34 mAbs detected a protein measuring about 100 to 110 kDa in KG1 cell lines and freshly isolated endothelial cells [42]. All these findings can explain the differences in size between the predicted protein and its native form. A band of the same size (approximately 110 kDa) was observed using new CD34 mAbs (clone 2E9) in canine leukemic cell lines, which were previously reported to be positive for CD34 [15]. Layton et al. [16] observed a specific band of 34 kDa with a novel SwCD34 antibody for the analysis of recombinant protein, which was produced in *E. coli* and used for immunization. The size of the detected protein agreed with the size of predicted recombinant swine CD34 protein. However, the authors did not analyze the swine CD34 in its native form presenting in corresponding tissue. On the other hand, a new ovine CD34 mAb (clone 8D11) was applied to enriched CD34 using magnetic sorting, as well as for the Western blot analysis of these cells. A specific band with a molecular weight of 100 kDa was observed [17]. Similarly, the newly produced boCD34ecf polyclonal antibody recognized several forms of bovine CD34, ranging from 105 to 150 kDa in different tissues (lymph node, spleen, bone marrow, lung, testis, muscle, brain, or liver), while the predicted molecular weight of bovine full-length CD34 measured 41 kDa, and the truncated form was about 35 kDa [38].

According to all analyses of hybridoma subclones presented in the current study, we eventually chose two subclones generated either against peptide 1 (mAb 58/47/26 and 58/47/34) or against recCD34 protein (mAb 182/7/80 and 575/36/8) for the final purification process. The purified mAbs were applied to SDS-PAGE analysis, resulting in the separation of these mAbs into heavy (50 kDa) and light (25 kDa) chains (Figure 12). In general, SDS-PAGE is a simple, relatively cheap, and widely used method for the analysis of antibody purity. Glycosylated heavy and light chains (of approximately 50 kDa and 25 kDa in size, respectively) basically arise from IgG class antibody samples under reducing conditions on SDS-PAGE analysis [44]. Moreover, we observed a relatively high immunoreactivity of purified mAb 58/47/26 and 58/47/34 to peptide 1, although a slightly lower immunoreactivity of these mAbs to recCD34 protein was noticed. On the other hand, purified mAb 182/7/80 and 575/36/8 exhibited high immunoreactivity to recCD34 protein (Figure 13). These findings agree with the results from Western blot analysis (Figure 11). Collectively, the observed immunoreactivity of purified mAbs decreased alongside the increased dilution ratio. Nevertheless, the ELISA method is commonly used to determine the immunoreactivity of antibodies at different concentrations [45]. Finally, we used purified mAbs to detect CD34^+^ cells in rabbit bone marrow (Figure 14). Up to 2–3% of positive BMMCs were recognized by all purified mAbs (58/47/26, 58/47/34, 182/7/80, and 575/36/8), which corresponded with the results obtained by the same hybridoma supernatants, as stated above. McSweeney et al. [15] successfully purified five canine CD34 mAbs (1H6, 2E9, 6B11, 5F11-3, and 5F11-6). Two of them (1H6 and 2E9) detected 2.1% ± 1.0% of cells in canine bone marrow. In our study, about 2% of the CD34^+^ cells also exhibited CD45 expression. The novel developed antibody clone 8D11 recognized 1.1% ± 0.4% of total sheep BMMCs and 3.7% ± 0.4% of total sheep cord blood cells, which were concurrently positive for CD45 [17]. Interestingly, the population of CD34^+^CD45^+^ cells detected in sheep peripheral blood using this antibody clone increased rapidly from 2.24% to 13.83% after two days of G-CSF blood mobilization. Thus, we here declare that newly produced and purified mAbs (58/47/26, 58/47/34, 182/7/80, and 575/36/8) can recognize either CD34^+^CD45^−^ or CD34^+^CD45^+^ cells in rabbit bone marrow, which are likely primitive hematopoietic stem cells and hematopoietic progenitors, respectively.

## 5. Conclusions

We have successfully generated and purified new mAbs which specifically recognize CD34 antigen in rabbit bone marrow or peripheral blood. Such reagents should be very useful in the field of veterinary research, as well as for regenerative medicine and cell therapy, since they can facilitate the establishment of a rabbit model for studying human hematological disorders. However, further research is needed to confirm the suitability of these antibodies for the isolation or enrichment of rabbit CD34^+^ cells with valuable stem cell characteristics.

## 6. Patents

The hybridoma cell lines and corresponding monoclonal antibodies specific to rabbit CD34 antigen are the exclusive property of the authors employed by NPPC-RIAP Nitra, and protected by utility models (no. PUV 50129-2024 and no. PUV 50130-2024).

## Data Availability

The datasets used and/or analyzed in the current study are available from the corresponding author upon reasonable request.

## Supplementary Materials

The following supporting information can be downloaded at: https://www.mdpi.com/article/10.3390/biom15071021/s1, Figures S1–S8: Western blot original images.

## Abbreviations

The following abbreviations are used in this manuscript:

Fc	Crystallizable fragments
mAbs	Monoclonal antibodies
HGPRT	Hypoxanthine guanine phosphoribosyltransferase
HAT	Xanthine-aminopterin-thymidine
NZW	New Zealand White
HB	Holic blue
NPPC-RIAP Nitra	NPPC—Research Institute for Animal Production Nitra
PBMCs	Peripheral blood mononuclear cells
BMMCs	Bone marrow mononuclear cells
recCD34	Recombinant rabbit CD34 protein
KLH	Keyhole limpet hemocyanin
DMEM	Dulbecco’s modified Eagle’s medium
ELISA	enzyme-linked immunosorbent assay
MSCs	mesenchymal stem cells
EPCs	endothelial progenitor cells
