# Supplementary figures and images for: Generation of Novel Monoclonal Antibodies Recognizing Rabbit CD34 Antigen
Source: Biomolecules. 2025 Jul 15;15(7):1021. doi: 10.3390/biom15071021 (PMC12294026; doi:10.3390/biom15071021)

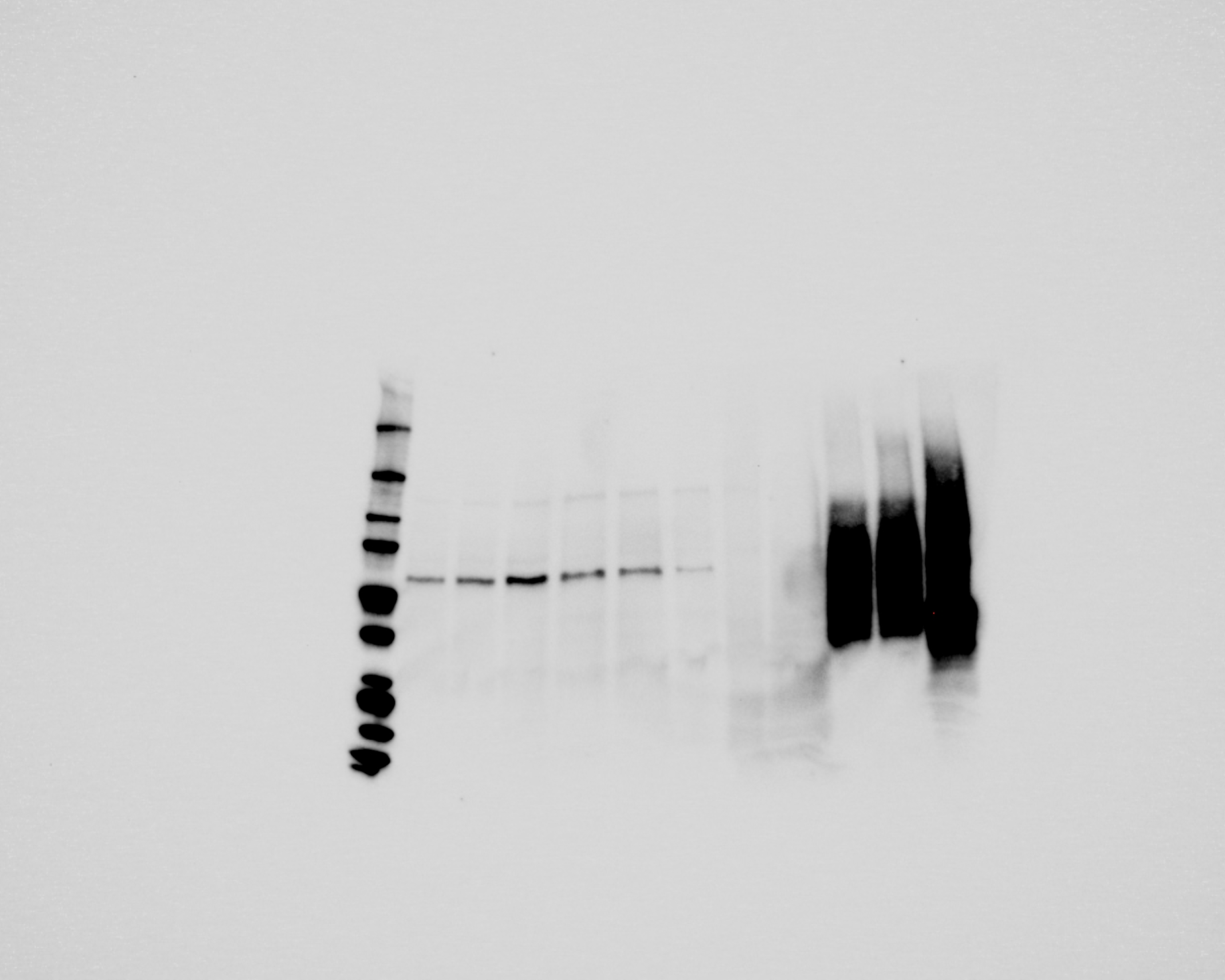

Supplement: Supplementary file 1 [file biomolecules-15-01021-s001.zip › Western blot original images /S3.jpg]

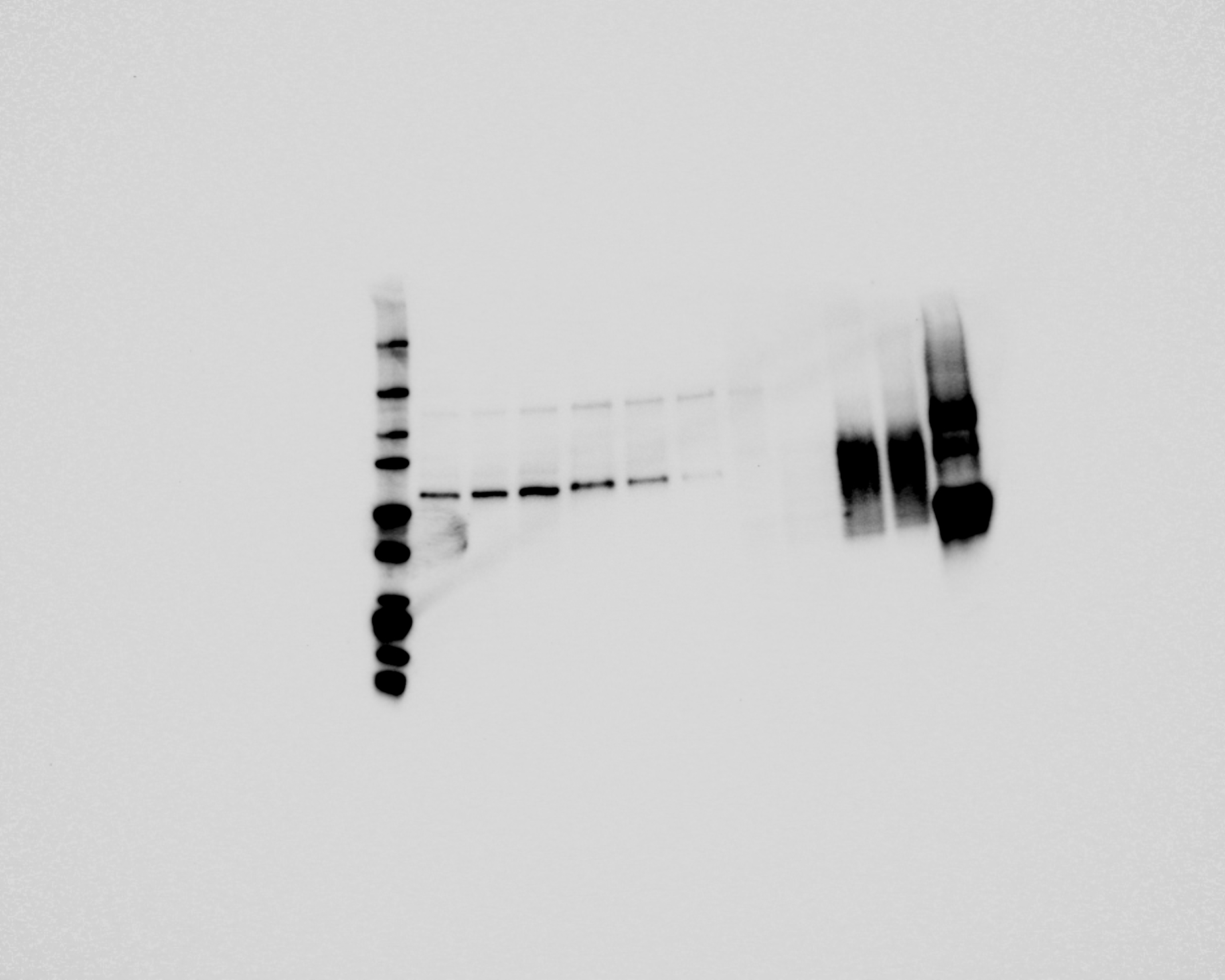

Supplement: Supplementary file 1 [file biomolecules-15-01021-s001.zip › Western blot original images /S4.jpg]

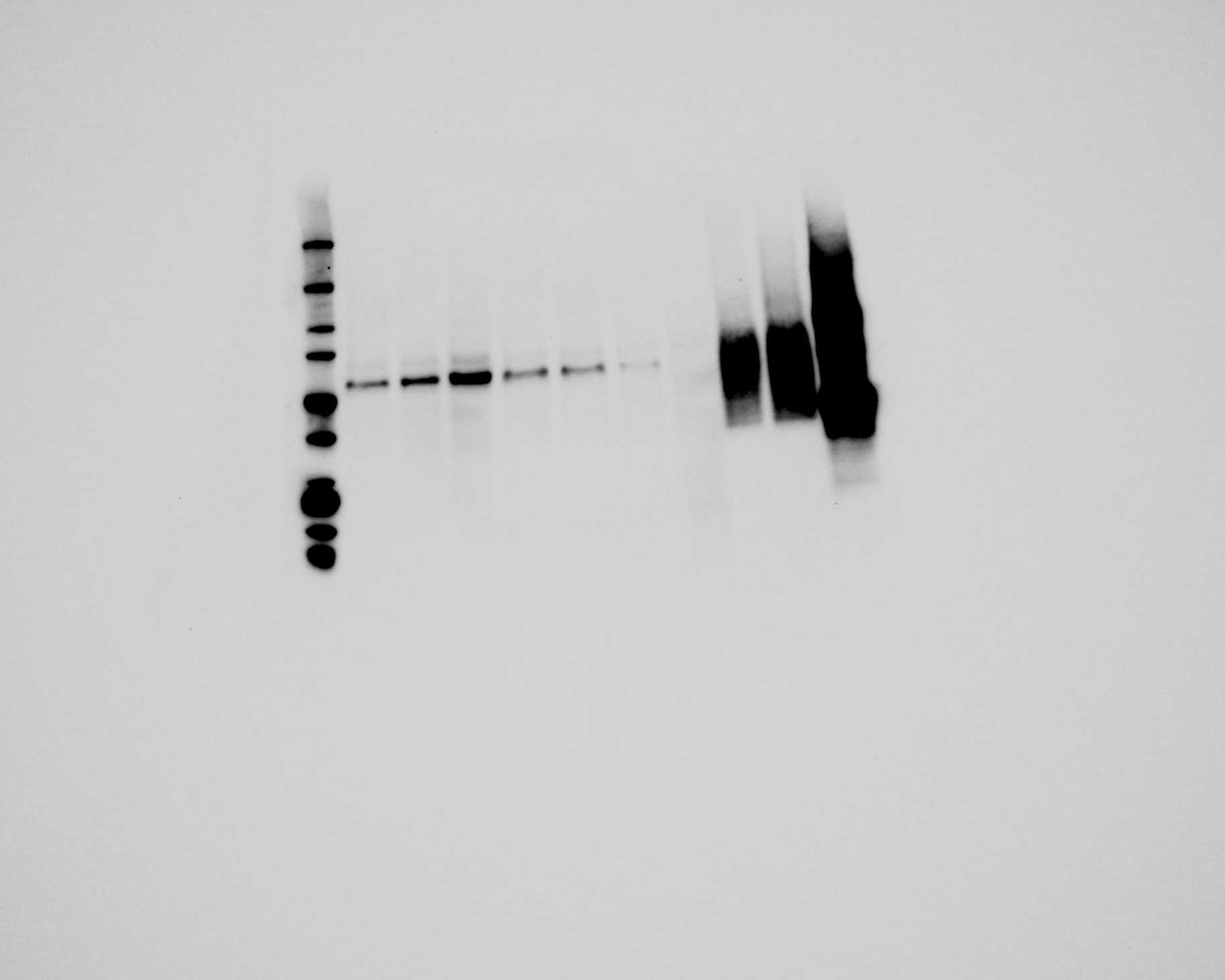

Supplement: Supplementary file 1 [file biomolecules-15-01021-s001.zip › Western blot original images /S5.jpg]

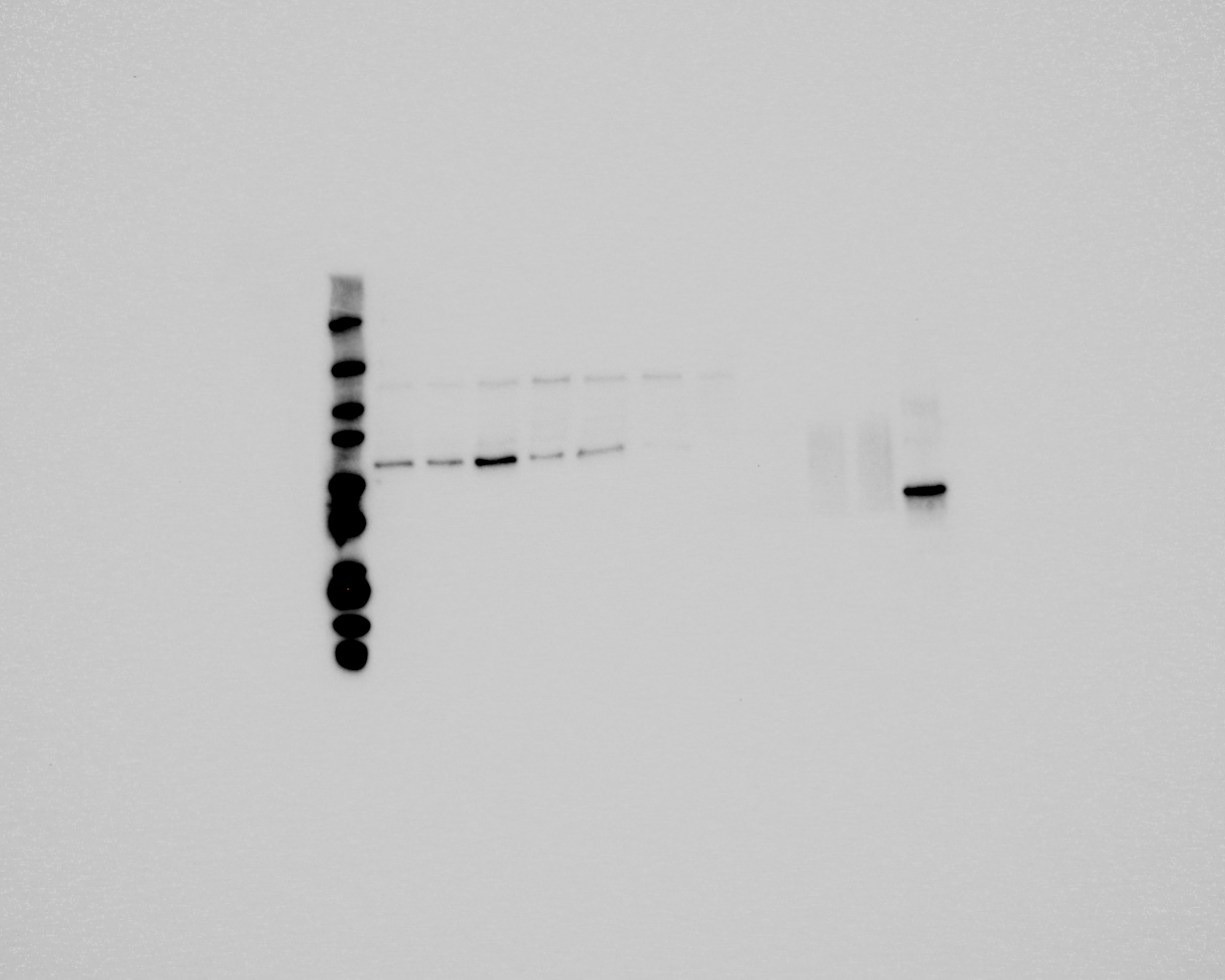

Supplement: Supplementary file 1 [file biomolecules-15-01021-s001.zip › Western blot original images /S7.jpg]

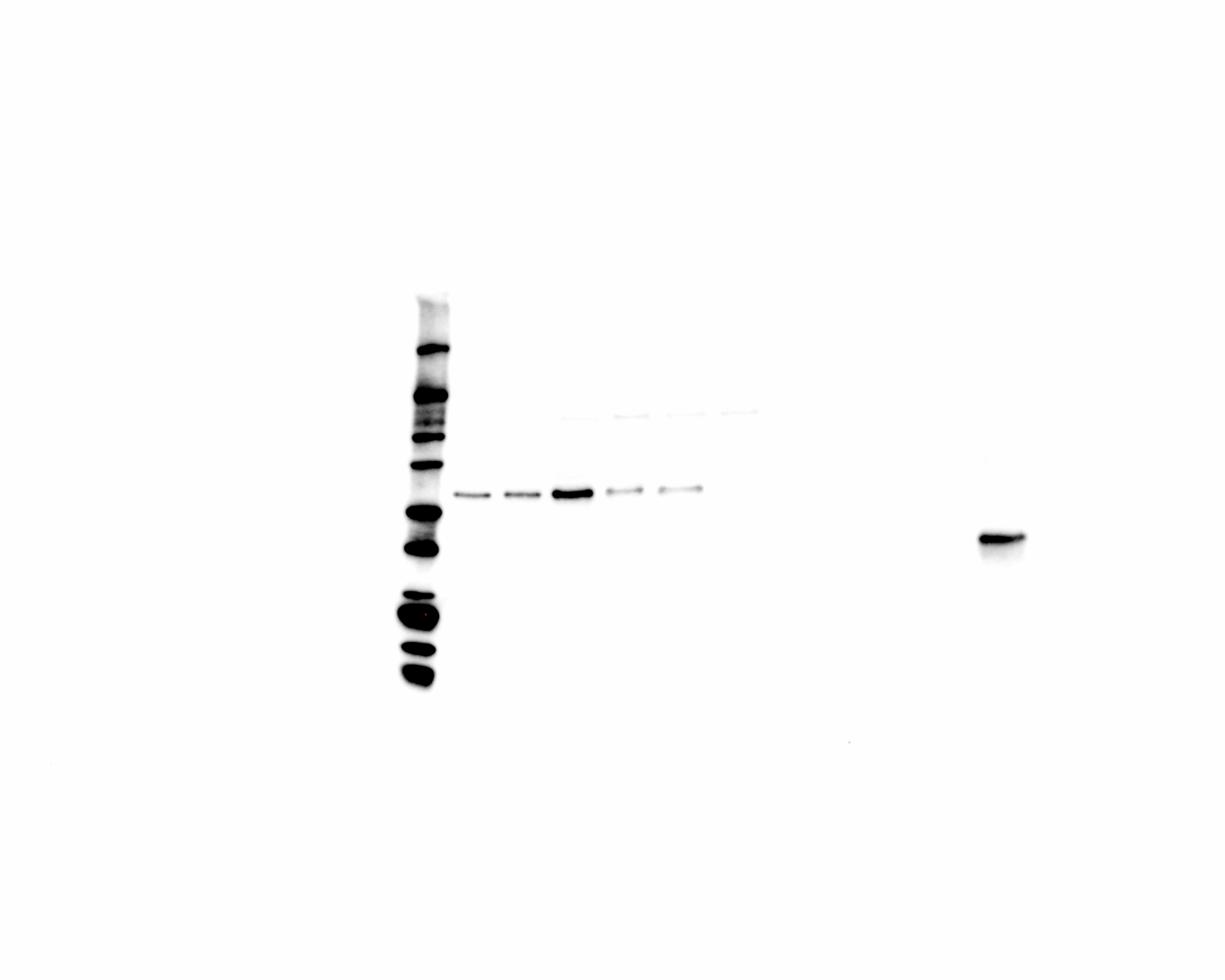

Supplement: Supplementary file 1 [file biomolecules-15-01021-s001.zip › Western blot original images /S8.jpg]

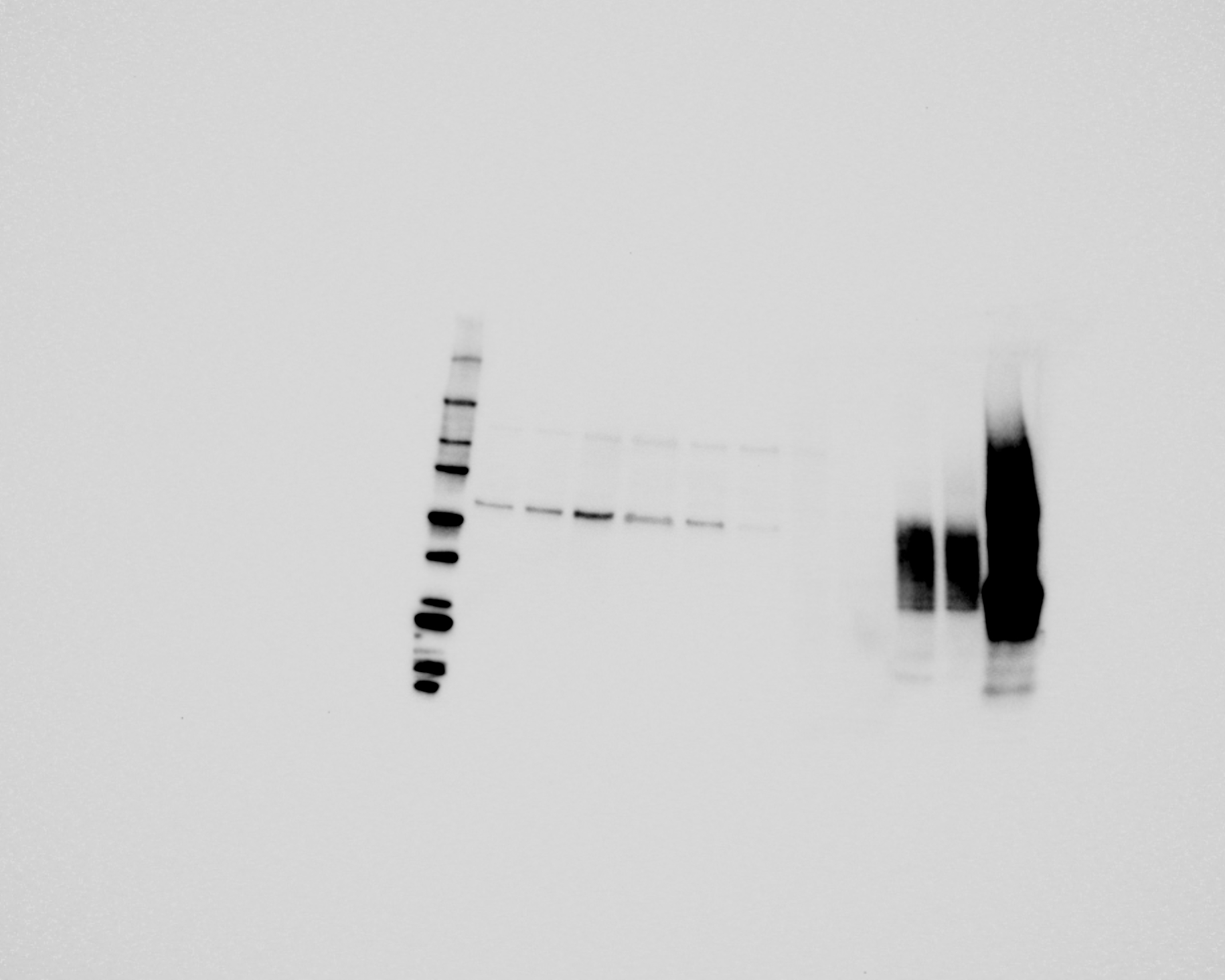

Supplement: Supplementary file 1 [file biomolecules-15-01021-s001.zip › Western blot original images /S6.jpg]

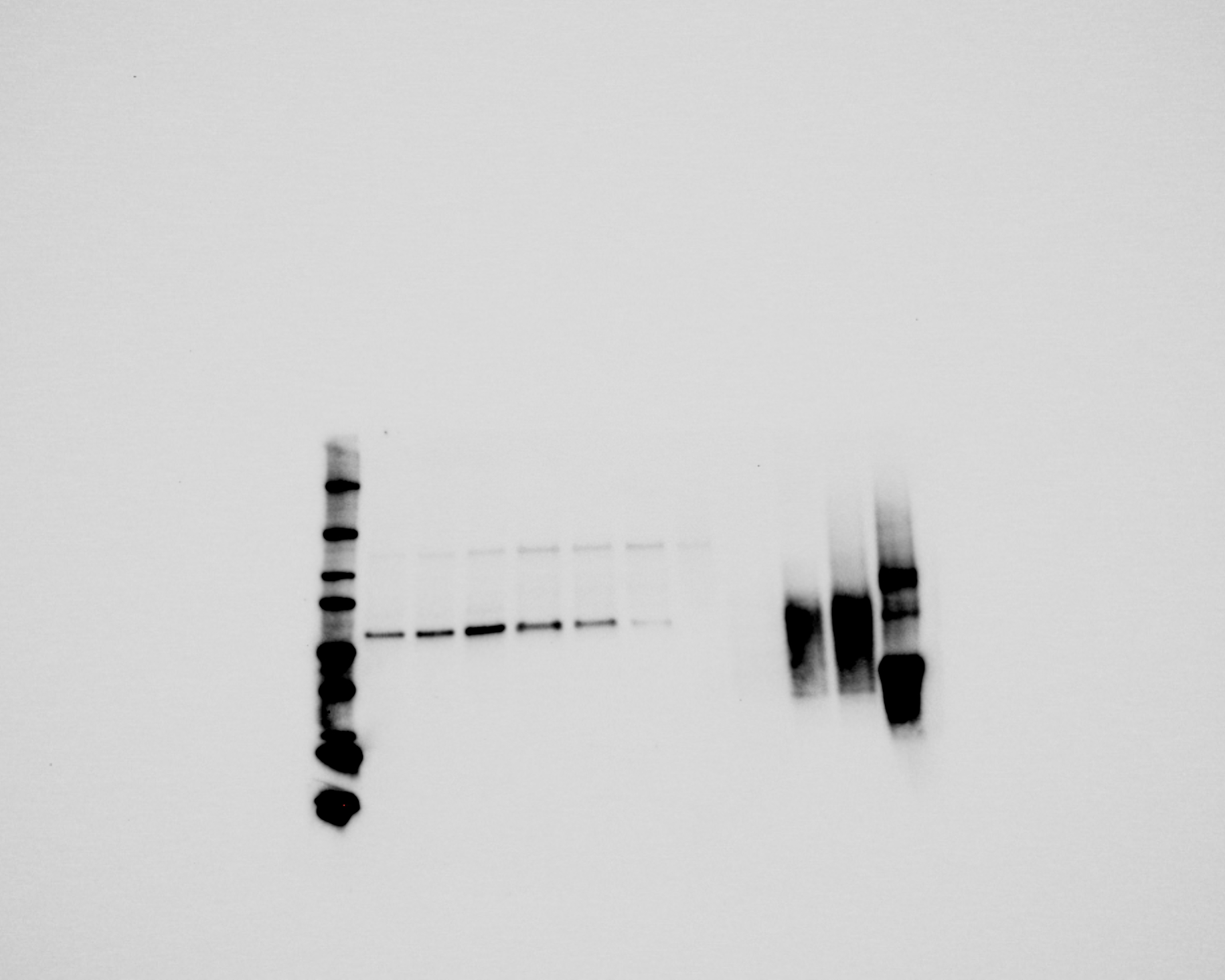

Supplement: Supplementary file 1 [file biomolecules-15-01021-s001.zip › Western blot original images /S2.jpg]

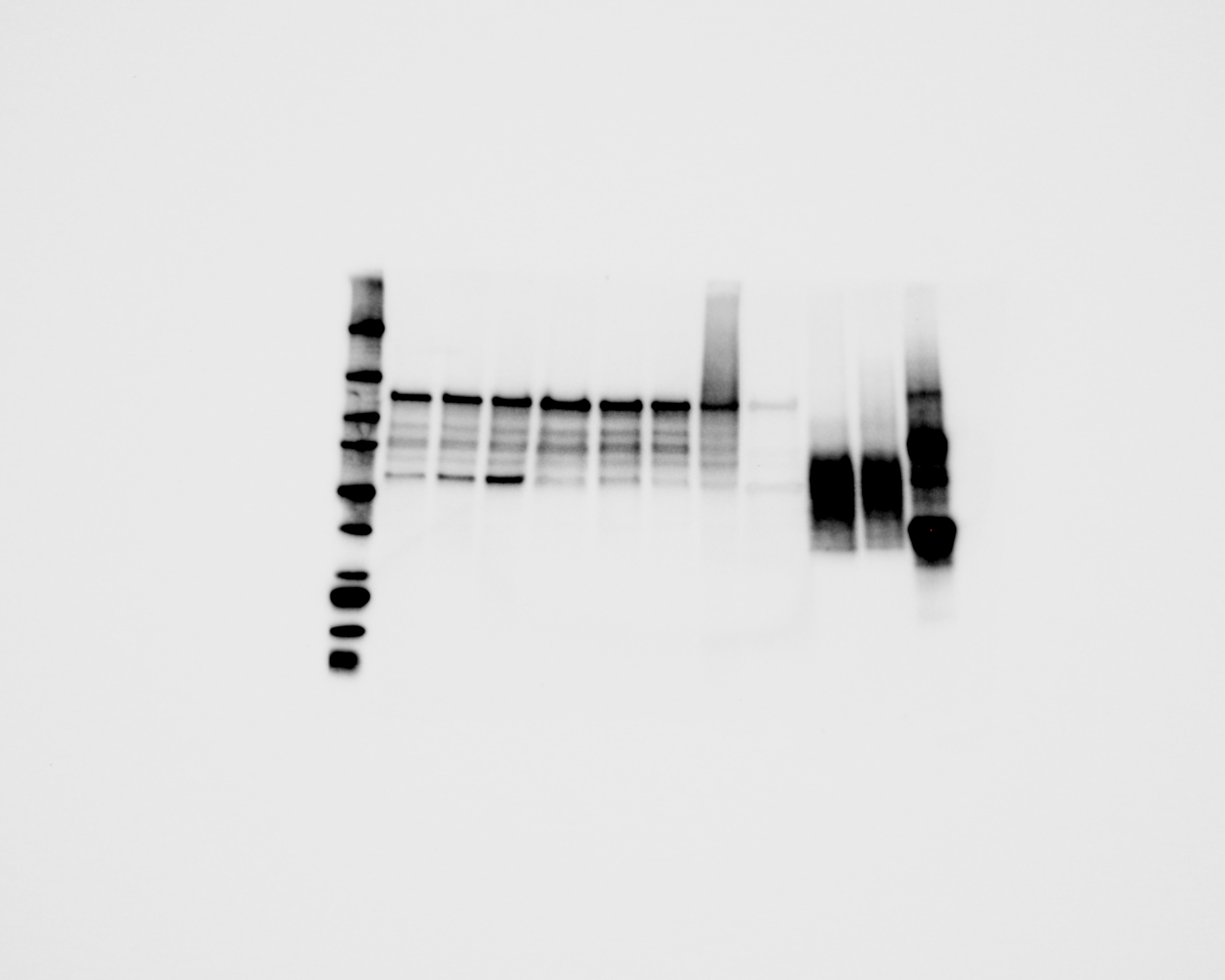

Supplement: Supplementary file 1 [file biomolecules-15-01021-s001.zip › Western blot original images /S1.jpg]
